# Controllability of structural brain networks

**DOI:** 10.1038/ncomms9414

**Published:** 2015-10-01

**Authors:** Shi Gu, Fabio Pasqualetti, Matthew Cieslak, Qawi K. Telesford, Alfred B. Yu, Ari E. Kahn, John D. Medaglia, Jean M. Vettel, Michael B. Miller, Scott T. Grafton, Danielle S. Bassett

**Affiliations:** 1 Department of Applied Mathematics and Computational Science, University of Pennsylvania, Philadelphia, Pennsylvania 19104, USA; 2 Department of Bioengineering, University of Pennsylvania, Philadelphia, Pennsylvania 19104, USA; 3 Department of Mechanical Engineering, University of California, Riverside, California 92521, USA; 4 Department of Psychological and Brain Sciences, University of California, Santa Barbara, California 93106, USA; 5 Translational Neuroscience Branch, Army Research Laboratory, Aberdeen Proving Ground, Aberdeen, Maryland 20783, USA; 6 Department of Electrical and Systems Engineering, University of Pennsylvania, Philadelphia, Pennsylvania 19104, USA

## Abstract

Cognitive function is driven by dynamic interactions between large-scale neural circuits or networks, enabling behaviour. However, fundamental principles constraining these dynamic network processes have remained elusive. Here we use tools from control and network theories to offer a mechanistic explanation for how the brain moves between cognitive states drawn from the network organization of white matter microstructure. Our results suggest that densely connected areas, particularly in the default mode system, facilitate the movement of the brain to many easily reachable states. Weakly connected areas, particularly in cognitive control systems, facilitate the movement of the brain to difficult-to-reach states. Areas located on the boundary between network communities, particularly in attentional control systems, facilitate the integration or segregation of diverse cognitive systems. Our results suggest that structural network differences between cognitive circuits dictate their distinct roles in controlling trajectories of brain network function.

## Abstract (web summary)

Cognitive control is fundamental to human intelligence, yet the principles constraining the neural dynamics of cognitive control remain elusive. Here, the authors use network control theory to demonstrate that the structure of brain networks dictates their functional role in controlling dynamics.

## Graphical abstract

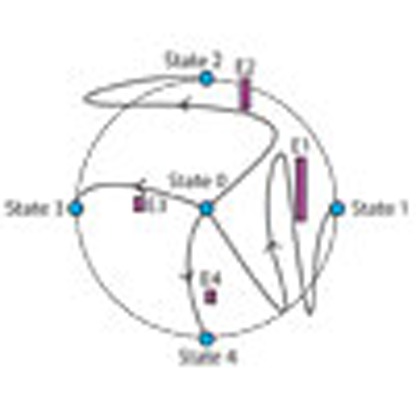

Neural systems alter their dynamics to meet task demands, enabling humans to perform the myriad complex cognitive functions necessary for everyday living. These capabilities are referred to as *cognitive control*^1^,^2^,^3^, and these include the ability to link multiple sources of information to solve problems, selective retrieval of information from memory, inhibition of inappropriate behavioural responses and active selection and maintenance of behaviourally relevant information online^4^. While traditional cognitive theories of control have implicated competitive dynamics in frontal cortices, fresh evidence from functional neuroimaging points to an account in which control functions rely on transitory changes in patterns of cooperation and competition between distributed neural systems, including regions in attention, default mode, frontoparietal and cingulo-opercular networks^4^.

Conceptually, cognitive control is analogous to mathematical notions of control used in engineering, where the state of a complex system can be modulated by energetic input. Networked systems—like the brain—are particularly interesting systems to control because of the role of the underlying architecture, which predisposes certain components to specific control actions. In the brain, neuronal ensembles or regions (nodes) are interlinked by anatomical wires (edges) in a complex architecture that has an impact on neural function^5^, development^6^, disease^7^ and rehabilitation^8^. It is plausible that the brain could regulate cognitive function by a transient network-level control process akin to those engineered in technological, social and cyberphysical systems. Yet, an exact understanding of the relationship between mathematical measures of controllability and notions of *cognitive control* from neuroscience remains elusive.

Here we capitalize on recent theoretical advances in *network control theory* to investigate how structural features of a brain network determine temporal features of cognitive dynamics (Fig. 1). We define a *trajectory* of a neural system to be the temporal path that the system traverses through diverse states, where a *state* is defined as the magnitude of neurophysiological activity across brain regions at a single time point. Controllability of a network refers to the possibility to manipulate network components to drive the system along a desired trajectory: that is a set of states culminating in a target state, chosen for its functional utility. We postulate that network controllability might be a mechanism of cognitive control: particular nodes (brain regions) at critical locations within the anatomical network act as drivers that move the system (brain) into specific modes of action (cognitive functions).

We exploit the network control theory to address two questions about how the large-scale circuitry of the human brain constrains its dynamics. First, is the human brain theoretically controllable? Growing evidence from brain computer interfaces^9^ and neuromodulation^10^ suggests that changes in regional activity (as measured using functional magnetic resonance imaging (fMRI) or electroencephalography) can alter the dynamics of brain function. We therefore hypothesize that the brain is theoretically *controllable* in the sense defined mathematically with the network control theory (see Methods). However, since many such dynamic processes have an impact on distributed neural circuits rather than single brain regions alone, we conjecture that the brain is difficult to control via localized interventions. Second, which areas of the brain are most influential in driving changes in brain state trajectories? We aim to directly test for a relationship between conceptual notions of cognitive control and the mathematical notions of network control in the context of known cognitive systems^11^.

To address these questions, we build structural brain networks from diffusion spectrum imaging (DSI) data acquired in triplicate from eight healthy human adults. We perform diffusion tractography to estimate the number of streamlines linking *N*=234 large-scale cortical and subcortical regions extracted from the Lausanne atlas^12^. We summarize these estimates in a weighted adjacency matrix whose entries reflect the number of streamlines connecting different regions. Finally, we perform a systematic study of the controllability of the dynamical network defined by the weighted adjacency matrix. This construction enables us to examine different controllability measures in individual participants and to demonstrate that structural network differences between cognitive circuits dictate their distinct roles in controlling trajectories of brain network function.

## Results

### Global controllability

We first sought to address the question: ‘Is the human brain theoretically controllable?'. Theoretical controllability is the first question that one asks of a system in the field of network control theory, as it provides a basic intuition for the ability of interventions to alter system states. In the context of the brain, this question amounts to asking: can the brain be moved into an arbitrary target state (for example, active memory retrieval versus mathematical calculations, or more generally health versus disease) by changing the activity of a single brain region? To answer this question, we evaluated the *global controllability*: the smallest eigenvalues of the controllability Gramian for each brain region as a control node. These values were consistently greater than 0, indicating that the system is theoretically controllable through a single region, but remained small (mean 2.5 × 10^−23^, standard deviation (STD) 4.8 × 10^−23^) with respect to the largest eigenvalues (always greater or equal to 1), indicating that in practice the system is extremely hard to control through a single region.

The minute nature of the values of global controllability motivates a more thorough examination of their reliability. In the Supplementary Methods, we show that values of the smallest eigenvalues of the Gramian are not reproducible across scanning sessions (see Supplementary Methods, where the *P* values for global controllability are consistently greater than 0.05). However, we do observe that the approximate order of these eigenvalues is reproducible and varies monotonically over spatial scales of the regional parcellation (Supplementary Methods). Together, these results indicate that, while the brain is consistently *theoretically controllable* through a single region, individual differences in global controllability cannot be accurately measured using these techniques.

### Regional controllability

We next sought to address the question: ‘which areas of the brain are most influential in constraining or facilitating changes in brain state trajectories?' To address this question, we employed three diagnostics of regional controllability: the average, modal and boundary controllability. Each of these diagnostics captures a different control goal^13^. *Average controllability* identifies brain areas that, on average, can steer the system into different states with little effort (that is, input energy). Here we define a *state* to be the vector of neurophysiological activity magnitudes across brain regions at a single time point. Loosely speaking, regions with high average controllability can move the brain to many easily reachable states. If control energy can be likened to cognitive effort and if brain states can be likened to cognitive functions, then these areas may be important in allowing the brain to move smoothly between many cognitive functions that require little cognitive effort. *Modal controllability* identifies brain areas that can push the brain into difficult-to-reach states (states that a require substantial input energy). From a cognitive perspective, these areas may be important in switching the brain between functions that require significant cognitive effort. *Boundary controllability* identifies brain areas that lie at the boundaries between network communities, controlling the integration of cognitive systems. From a cognitive perspective, these areas may be important in gating, synchronizing or otherwise manipulating information across different cognitive processes, such as audition and language, or vision and motor. For mathematical definitions of these diagnostics, see Methods.

### Average controllability

**Figure 2:**
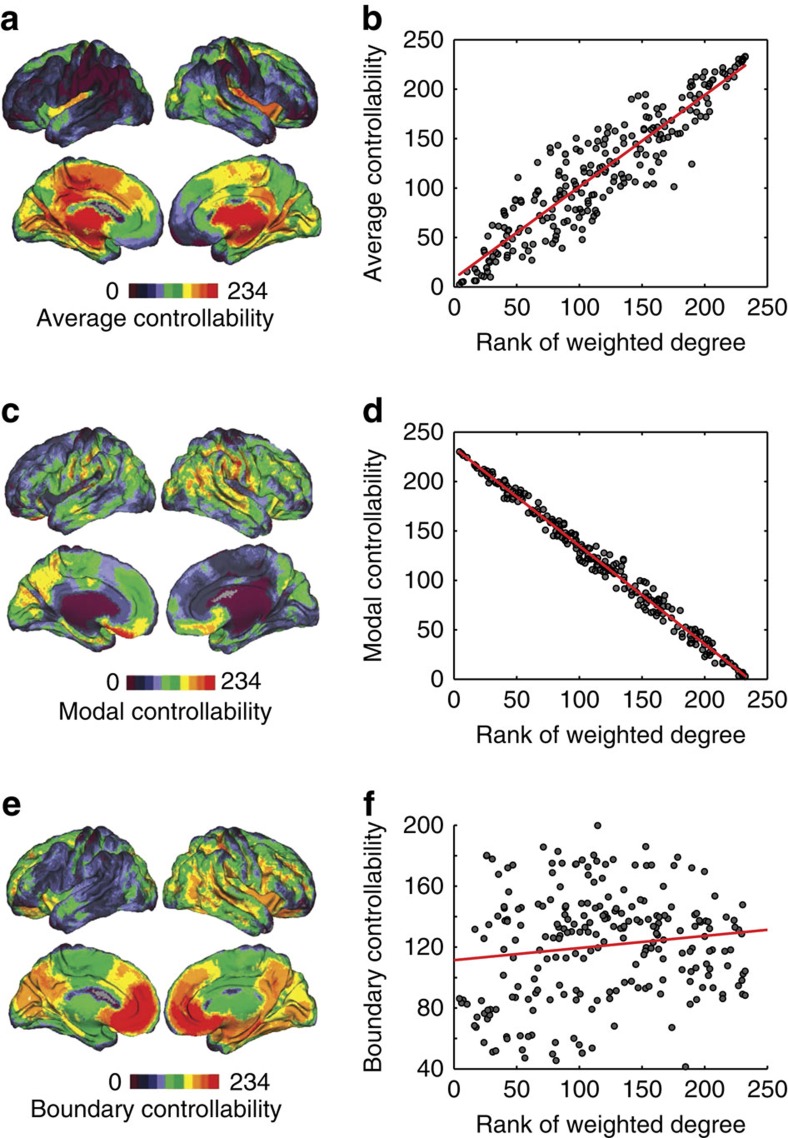
Brain network control properties. (**a**) Average controllability quantifies control to many easily reached states. Here we show controllability values, averaged across three scanning sessions and eight persons, and ranked for all 234 brain regions plotted on a surface visualization. Warmer colours indicate larger values of average controllability. (**b**) Scatter plot of weighted degree (ranked for all 234 brain regions), averaged across three scanning sessions and eight persons, versus average controllability (Pearson correlation *r*=0.91, *P*=8 × 10^−92^). (**c**) Modal controllability quantifies control to difficult-to-reach states. Here we show modal controllability values, averaged across three scanning sessions and eight persons, and ranked for all 234 brain regions plotted on a surface visualization. (**d**) Scatter plot of weighted degree (ranked for all 234 brain regions), averaged across three scanning sessions and eight persons, versus modal controllability (*r*=−0.99, *P*=2 × 10^−213^). (**e**) Boundary controllability quantifies control to decouple or integrate network modules. Here we show boundary controllability values, averaged across three scanning sessions and eight persons, and ranked for all 234 brain regions plotted on a surface visualization. (**f**) Scatter plot of weighted degree (ranked for all 234 brain regions), averaged across three scanning sessions and eight persons, versus boundary controllability (*r*=0.13, *P*=0.03). In **a**,**c**,**e**, warmer colours indicate larger controllability values, which have been averaged over both replicates (three scanning sessions) and eight subjects. These results are reliable over a range of atlas resolutions and are consistent with findings using a network composed of only cortical circuitry (see Supplementary Methods). Note that nodes are sorted in an ascending order of the weighted degree.

Average controllability identifies brain areas that can steer the system into many different states, or patterns of neurophysiological activity magnitudes across brain regions. The average controllability is greatest in precuneus, posterior cingulate, superior frontal, paracentral, precentral and subcortical structures (Fig. 2a). Strikingly similar to the structural ‘core' of the human cerebral cortex^12^, these regions are ‘hubs', having high network degree defined as the average weight of edges emanating from that region. Indeed, the average controllability is strongly correlated with weighted degree (also known as *node strength*; Pearson correlation *r*=0.91, *P*=8 × 10^−92^; Fig. 2b). In addition to being structural hubs, we note that these regions also form the anterior and posterior medial portions of the default mode system (which we explicitly test in the following section).

### Modal controllability

Modal controllability identifies brain areas that can steer the system into difficult-to-reach states. The modal controllability is greatest in postcentral, supramarginal, inferior parietal, pars orbitalis, medial orbitofrontal and rostral middle frontal cortices (Fig. 2c). Areas with high modal controllability are not hubs of the network but instead have low degree. The modal controllability is strongly anticorrelated with weighted degree (Pearson correlation *r*=−0.99, *P*=2 × 10^−213^; Fig. 2d), consistent with the notion that difficult-to-reach states require the control of sparsely connected areas.

### Boundary controllability

Boundary controllability identifies brain areas that can steer the system into states where different cognitive systems are either coupled or decoupled. This control goal complements but differs from those of average and modal controllability. The boundary controllability is greatest in rostral middle frontal, lateral orbitofrontal, frontal pole, medial orbitofrontal, superior frontal and anterior cingulate cortices (Fig. 2e). In contrast to areas with high average or modal controllability, areas with high boundary controllability are neither hubs nor non-hubs. The boundary controllability of all brain regions is not strongly correlated or anticorrelated with weighted degree (Pearson correlation *r*=0.13, *P*=0.03; Fig. 2f).

### Reliability of controllability diagnostics

With any new technique, it is critical to evaluate the reliability of the estimated diagnostics. The regional controllability diagnostics that we report and utilize here are highly reliable across multiple scanning sessions (see Supplementary Methods), indicating their potential use in explaining individual differences in cortical function. Moreover, the anatomical distribution of controllability diagnostics is consistent across five parcellation schemes segregating the brain into 83, 129, 234, 463 and 1,015 regions of interest (see Supplementary Methods), suggesting that these measures are robust quantifications of brain dynamics.

**Figure 3:**
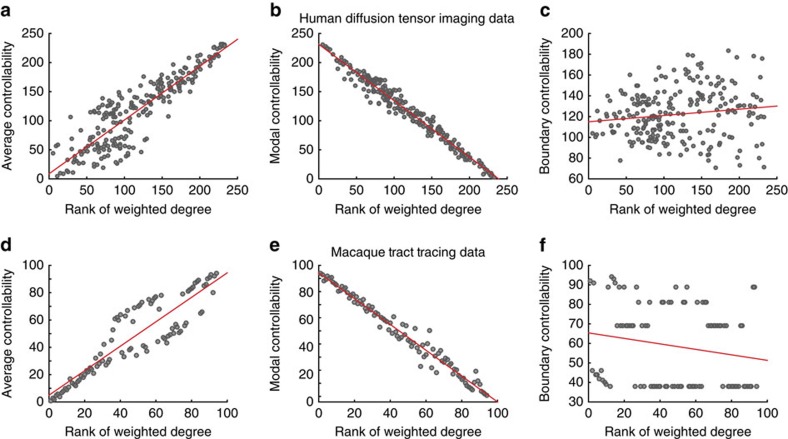
Reliability and conservation of brain network control properties. Brain network control properties are reliable across imaging acquisition and are conserved in non-human primates. Scatter plots of weighted degree (ranked for all 234 brain regions) versus (**a**,**d**) average controllability (Pearson correlation coefficient *r*=0.88, *P*=1.0 × 10^−78^; *r*=0.90, *P*=4.9 × 10^−34^), (**b**,**e**) modal controllability (*r*=−0.99, *P*=3.9 × 0^−179^; *r*=−0.99, *P*=1.3 × 10^−72^) and (**c**,**f**) boundary controllability (*r*=0.14, *P*=0.028; *r*=−0.19, *P*=0.074) for (**a**–**c**) human diffusion tensor imaging data and (**d–f**) macaque tract tracing data. In **a**–**c**, controllability values are averaged over 85 subjects.

In addition to reliability across spatial resolutions and multiple scanning sessions, we next asked whether our results could be reliably reproduced using different imaging acquisitions and different subject cohorts. To address this question, we constructed 234-region structural brain networks from diffusion tensor imaging data acquired on an independent sample of 85 healthy human adult subjects^14^,^15^ (see the Supplementary Methods for details on demographics, acquisition, preprocessing and tractography). Consistent with our previous results, these data display a strong positive correlation between average controllability and weighted degree (Pearson correlation coefficient *r*=0.88, *P*=2.5 × 10^−80^; see Fig. 3a), a strong negative correlation between modal controllability and weighted degree (*r*=−0.99, *P*=1.2−10^−184^; see Fig. 3b), and a weaker relationship between boundary controllability and weighted degree (*r*=0.0084, *P*=0.90; see Fig. 3c). These data support the claim that the architecture of structural brain networks differentially has an impact on the putative role of brain regions in different control strategies.

### Conservation across species

Finally, we asked whether the relationship between controllability and topology was conserved in non-human primates. Using a data set drawn from CoCoMac^16^ that delineated 2,402 projections between 95 cortical and subcortical areas^17^, we again observed consistent results, including a strong positive correlation between average controllability and weighted degree (Pearson correlation coefficient *r*=0.90, *P*=4.9 × 10^−34^; see Fig. 3d), a strong negative correlation between modal controllability and weighted degree (*r*=−0.99, *P*=1.3 × 10^−72^; see Fig. 3e) and a nonsignificant correlation between boundary controllability and weighted degree (*r*=−0.19, *P*=0.074; see Fig. 3f). These data indicate that the role of brain hubs and non-hubs in different control strategies is conserved across human and non-human primates.

### Regional controllability of cognitive systems

After confirming reliability and conservation of our findings, we asked the question ‘are control regions differentially located in or between known cognitive systems?' Drawing from the literature, we formulate three specific hypotheses addressing this question. First, on the basis of the fact that average controllability identifies areas of the brain that may be important in steering the system into many easily reachable states, we hypothesize that areas of high average controllability would map on to areas active in the brain's baseline or ‘default' state (the resting state), from which the brain smoothly moves to multitudinous task states. In contrast, modal controllability identifies areas of the brain that may be important in steering the system to difficult-to-reach states. We hypothesize that areas of high modal controllability would therefore map on to areas responsible for the brain's transitions between difficult tasks, specifically executive areas involved in cognitive control. Finally, boundary controllability identifies areas of the brain that can steer the system into states where different cognitive systems are either decoupled or integrated. Because these areas mathematically sit at the boundaries between network communities or putative functional modules, we expect that these areas would map relatively uniformly on all cognitive systems: each system having a few boundary nodes that might play a role in linking that system to another. However, we also postulate a particular enrichment of the attention systems, on the basis of their role in feature selection, gating, orienting and multitasking, which constrain integration across other cognitive systems.

To test these hypotheses, we assigned the 234 regions of the Lausanne atlas to the following large-scale cortical networks, which we refer to as ‘cognitive systems': auditory, visual, sensorimotor, ventral attention, dorsal attention, default mode, frontoparietal and cingulo-opercular. This set of cognitive systems, and the association of regions to these cognitive systems, has previously been extracted from resting state data using a network-based clustering approach^11^ and has been widely applied to examine the roles of cognitive systems in task-based and resting-state connectivity^18^ (see the Supplementary Methods for regional attributions to systems).

**Figure 4:**
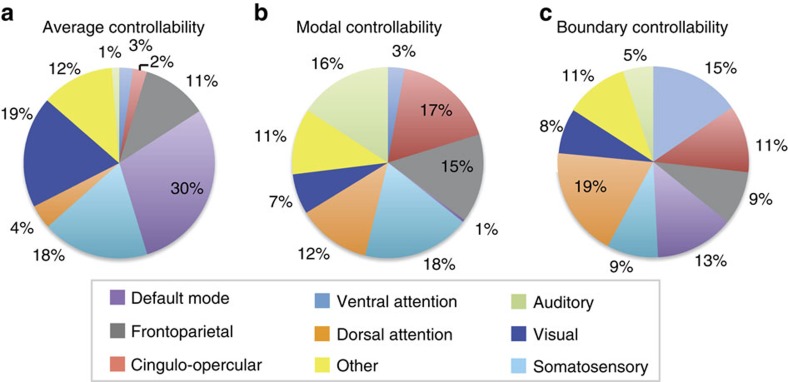
Control roles of cognitive systems. Cognitive control hubs are differentially located across cognitive systems. (**a**) Hubs of average controllability are preferentially located in the default mode system. (**b**) Hubs of modal controllability are predominantly located in cognitive control systems, including both the frontoparietal and cingulo-opercular systems. (**c**) Hubs of boundary controllability are distributed throughout all systems, with the two predominant systems being ventral and dorsal attention systems. Control hubs have been identified at the group level as the 30 regions with the highest controllability values (averaged over three replicates and eight subjects). Raw percentages of control hubs present in each system have been normalized by the number of regions in the cognitive system. By applying this normalization, systems composed of a larger number of regions have the same chance of housing one of the top 30 control hubs as systems composed of a smaller number of regions.

We find that regions of high controllability are differentially associated with the eight cognitive systems (Fig. 4), suggesting that different cognitive systems play different control roles. We define the set of high control hubs as the 30 regions with the largest controllability values (averaged over all scans), and we calculate the per cent of hubs present from each of the eight cognitive systems. To correct for system size, we normalize the raw percentage of hubs present in a given cognitive system by the number of regions in a cognitive system. By applying this normalization, systems composed of a larger number of regions do not have an increased normalized probability of housing one of the top 30 control hubs than systems composed of a smaller number of regions. Consistent with our hypotheses, 30% of average control hubs lie in the default mode system, 32% of modal control hubs lie in the frontoparietal and cingulo-opercular cognitive control systems and 34% of boundary control hubs lie in the ventral and dorsal attention systems. Our results are qualitatively similar if we choose a larger or smaller set of control hubs, in different imaging acquisition schemes including diffusion tensor imaging, and in a large independent subject cohort (see Supplementary Methods).

**Figure 5:**
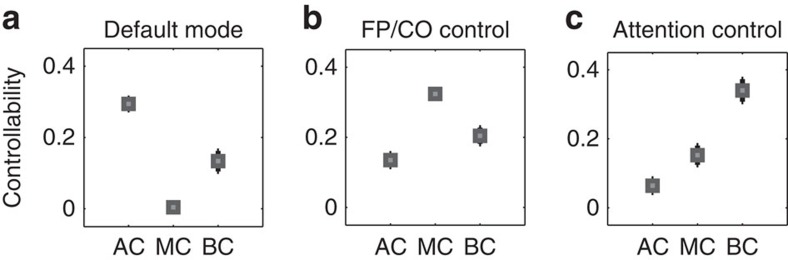
Differential recruitment of cognitive systems to network control. Average controllability (AC), modal controllability (MC) and boundary controllability (BC) hubs are differentially located in default mode (**a**) frontoparietal and cingulo-opercular cognitive control (**b**) and attentional control (**c**) systems. Values are averaged over the three replicates for each of eight subjects; error bars indicate s.d. of the mean over subjects.

These results suggest the presence of a controllability-by-system interaction: certain types of controllability may be utilized or enabled by different cognitive systems. To directly test for this interaction, we extract control hubs for each scan, determine their association with the three hypothesized control systems (default mode, frontoparietal and cingulo-opercular cognitive control, and attentional control) and quantify the mean controllability value for all hubs in each system (Fig. 5). We observe that regions of the default mode system form strong average controllability hubs but weaker modal and boundary controllability hubs. Regions of the cognitive control networks (frontoparietal and cingulo-opercular) form strong modal controllability hubs and regions of the attentional control networks (ventral and dorsal) form strong boundary controllability hubs. To statistically validate this finding, we perform a repeated measures two-way analysis of variance with cognitive system and controllability diagnostic as categorical factors, and with scan replicate as a repeated measure. The main effect of system is significant (*F*(9)=42.40, *P*=0); the main effect of diagnostic is significant (*F*(2)=22.25, *P*=0.0013); and the interaction between system and diagnostic is also significant (*F*(18)=39.81, *P*=0). These statistics indeed suggest that structural differences between the default mode, cognitive control and attentional control systems may facilitate their distinct roles in controlling trajectories of brain network function. From a cognitive perspective, these results suggest that (i) default mode areas may be important in allowing the brain to move smoothly between many cognitive functions that require little cognitive effort, (ii) frontoparietal and cingulo-opercular areas may be important in switching the brain between functions that require significant cognitive effort and (iii) attention areas may be important in gating, synchronizing or otherwise manipulating information across different cognitive processes. Importantly, these results are robustly observed in different imaging acquisition schemes including both with diffusion tensor imaging and in a large independent subject cohort (see Supplementary Methods).

## Discussion

The brain is a networked dynamical system that moves between diverse cognitive states to enable complex behaviours. Fundamental principles constraining these trajectories have remained elusive. Here we use network control theory to offer a mechanistic explanation for how the brain moves between cognitive states on the basis of white matter microstructure. Densely connected areas are postulated to facilitate the movement of the brain to many easily reachable states and are preferentially located in the default mode system. Weakly connected areas, predominantly located in cognitive control systems, are postulated to facilitate the movement of the brain to difficult-to-reach states. Areas at the boundary between network communities, predominantly located in attentional control systems, are postulated to facilitate the integration or segregation of cognitive systems. This body of work suggests that structural network differences between the default mode, cognitive control and attentional control systems dictate their distinct roles in brain network function.

Network control theory predicts the controllability of large-scale neural circuitry. The smallest eigenvalues of the controllability Gramian suggest that structural brain network architecture is controllable, but is not *easily* controllable. The possibility of control is consistent with studies demonstrating that (i) lesions to single brain areas can have an impact on neural activity, connectivity and human behaviour^19^ and (ii) subjects can control regional activity to modulate pain perception^20^. Yet, the brain cannot be easily controlled: it is practically impossible to move the brain to *any* target state that we might desire with little control action (see Supplementary Note 1). For example, moving any diseased state to a healthy state is difficult, even with a complex combinations of drugs, brain stimulation and cognitive therapies^21^. This control difficulty illustrates the complexity of cognitive function and calls for the development of new tools to determine which trajectories are amenable to control, informing targeted therapies including brain stimulation^22^.

Average controllability is posited to quantify a node's role in moving the system to many easily reachable states. We show that brain regions with high average controllability tend to be highly connected hubs, located predominantly in anterior and posterior medial portions of the default mode system. Other portions of this system may be important in other control strategies. This suggests that the brain has a baseline resting state organization that is optimized to allow the brain to move to a large number of easily reachable states. If we assume that the brain has been optimized over evolutionary timescales to enable a complex functional battery^23^, these results suggest the intriguing possibility that the large majority of functions performed by the brain are easily reachable from the default mode state. Complementing prior work demonstrating that the default mode is activated during ‘rest' and largely deactivated during many task conditions^24^, our data suggest that the default mode is a pluripotent ‘ground state', which can move the brain into many task-based activation profiles (‘excited states'^25^). Moreover, the default mode is the state to which the brain relaxes back after the task has been performed, readying the brain to move to new task states, when the cycle will repeat. Importantly, these dynamic notions of brain function are predicated on the underlying structure of the white matter pathways facilitating cognitive processes.

Our observations may be complemented by work highlighting features of brain network hubs that might contribute to the dynamic functional role outlined by network control theory. The so-called *rich-club* organization of the human connectome^26^ refers to the fact that many brain network hubs are densely interconnected to one another^27^. This organization is evident across species^26^,^28^, changes over development^6^ and is altered in disease^29^,^30^. The rich club is thought to play important roles in information integration^26^, facilitating the functional dynamics necessary for cognitive functions. The fact that hubs in many different cognitive systems are linked together potentially provides a structural substrate for the movement of the brain between cognitive processes. Supporting this hypothesis, work by Senden *et al*.^31^ uses a spin glass model of neural networks for simulating stable configurations of cortical activity and shows that networks with rich-club architecture display functional dynamics characterized by a larger set of attractors (and hence greater diversity of the functional repertoire) than that expected in scale-free networks devoid of rich clubs. Our results provide a theoretical mechanism for these empirical findings: hub nodes in the brain tend to have high average controllability, indicating that they are critical for moving the brain into many easily reachable states (attractors), thereby facilitating a great diversity of functional dynamics.

The fact that structural hubs, particularly in the default mode network, play such a striking role in brain network controllability may further help to explain the growing body of evidence indicating that disease states can preferentially target hub areas^7^,^32^. *In silico* studies suggest that lesions to highly structurally connected areas have a greater impact on ensuing functional connectivity than lesions to sparsely connected areas^33^. Moreover, alterations to default mode hubs are associated with drastic changes in cognitive function associated with normative aging^34^ and neurodegenerative disorders such as Alzheimer's disease^35^. Our results provide a mechanistic explanation for these findings by suggesting that hubs form the key control points in brain networks; alterations to hub regions can therefore have disproportionately high impacts on system function.

While our results demonstrate that hubs are theoretically implicated in moving the brain to many easily reachable states, weakly connected areas are critical for moving the brain to difficult-to-reach states. We observe that these modal control points tend to be predominantly located in cognitive control systems including the frontoparietal and cingulo-opercular networks. These two systems are characterized by different functional connectivity patterns at rest^11^ and are thought to support distinct functional roles^36^: task-switching and task-set maintenance. Our results suggest a fundamental underlying mechanism of cognitive control: brain regions sparsely interconnected with the rest of the brain are critically important for moving the system into difficult-to-reach states. This theoretical hypothesis is consistent with the increased engagement of the cognitive control system in highly effortful tasks^37^.

More generally, the fact that weak connections play a critical role in system dynamics is one that has traditionally received little attention. However, recent work demonstrates the relevance of weak connections for cognitive function and psychiatric disease. For example, the topology of weak connections in resting state fMRI has been used to classify healthy volunteers versus schizophrenia patients^38^. Moreover, the topology of weak connections more accurately correlates with intelligent quotients than the topology of strong connections^39^,^40^. These findings challenge the traditional view that strong connections alone are critical for brain dynamics. Our results provide a mechanistic rationale for the importance of weak connections, which are theoretically critical in enabling a system to move to difficult-to-reach states, including high-performance states (measured by IQ) or altered performance states (observed in psychiatric conditions).

In addition to the two mechanisms that enable trajectories to many easily reachable states and a few difficult-to-reach states, networked systems often utilize a third mechanism—boundary controllability—that enables the segregation or integration of network modules. Modular structure has been reported in structural, functional and dynamic brain networks^41^. In resting state connectivity studies, these modules have been linked to known cognitive systems^11^. Our results suggest that a widely distributed set of brain areas across all of these systems enables segregation and integration of putative cognitive modules. We also observe an enrichment of boundary control hubs in dorsal and ventral attentional systems, suggesting that attentional control may be implemented by boundary control strategies integrating or segregating disparate cognitive systems. Such a theoretical prediction is supported by evidence that attentional control integrates different cognitive functions^3^, and that disconnection of attentional networks is accompanied by extensive cognitive deficits^42^.

Finally, it is important to address methodological considerations. Graph theory has proven to be an extremely productive framework in which to understand the structure and function of large-scale brain circuits^5^ and their implications for human cognition^43^; alternative approaches that build on this framework—such as network control theory—necessarily require sceptical evaluation to clearly delineate value added. Graph theory specifically and network science more generally have provided a toolbox of diagnostics to describe the organization of graphs or networks. Yet, the relationships between this organization and the system's function remain speculative at worst and correlative at best. Groundbreaking new discoveries will necessitate a fundamental turn from descriptive statistics towards mechanistic predictions. What are the mechanisms by which network structure affects functional dynamics? Moreover, how could one intervene in a network to push the system dynamics towards a specific, targeted goal? To address these questions, we must have a framework that incorporates not just brain network structure but also models neural dynamics. Network control theory offers exactly such a framework, along with a toolbox for selecting control nodes to effect specific control strategies (for example, average, modal and boundary). In the context of this study, the advantages are clear: using graph theory, we can identify regions of high (low) degree, while using network control theory, we can understand the functional role of these regions as being critical for guiding the movement of the brain into many easy-to-reach (difficult-to-reach) states. More generally, network control theory offers invaluable theoretically validated tools to inform explanations of brain function (for example, cognitive processes and computations), perturbations of brain function (via non-invasive stimulation paradigms) and predictions of brain function (for example, in altered or engineered neural architectures).

Decades of research demonstrate that neural dynamics are *nonlinear*. Yet, our approach is built on a linear model of these dynamics, and it is therefore imperative to delineate its strengths and weaknesses. First, we note that nonlinear behaviour may be accurately approximated by linear behaviour in certain scenarios (see, for example, ref. 44). Indeed, ref. 45 proposes a *linearized* model for the nonlinear neural dynamics described in ref. 44, and ref. 44 shows that predictions of function from structure can be obtained with both linear and nonlinear models. Second, we note that the controllability of a linearized model has implications for the controllability of a nonlinear model: if the linearized system is controllable, then the nonlinear system is *locally controllable*^46^,^47^. Third, linear models of a system accurately approximate nonlinear models in a neighbourhood of the operating point. For example, in *gain scheduling*, linear controllers are used to control a nonlinear system: each controller is designed on the basis of a linearization of the system around an operating point^48^, and an observable parameter is used to switch between controllers. Gain scheduling has been successful in many different application areas, including flight and process control, proving that controllers based on linearized dynamics can be effectively used for the control of nonlinear dynamics. Thus, while neural dynamics are inherently nonlinear, the study of linear models of neural dynamics can offer fundamental insights into system function.

Our approach is also built on diffusion imaging data and associated tractography methods. Important limitations of current tractography algorithms include (i) the inability to determine the precise origin/termination of connections, (ii) difficulty in distinguishing branching from merging or kissing axons and (iii) inability to distinguish afferents from efferents^49^. These limitations motivate ongoing methodological development^50^ in combination with post-mortem validation^51^ and constrain interpretations. Here we have used DSI data^52^ acquired with 257 directions using a *Q*5 half-shell acquisition scheme, and on which we applied a *q*-space diffeomorphic reconstruction^53^ (see Supplementary Methods). The tracking parameters used here were also used in ref. 54, which shows them to produce repeatable connectomes within and between individuals. These parameters were chosen because they are relatively conservative and accurately produced known fascicles while minimizing spurious streamlines. This approach provides significantly more data for tractography than the more common 30-direction diffusion tensor imaging (DTI) acquisition, particularly in estimates of longer fibres, and fibres located away from the medial wall. In the future, should accurate estimates of directionality be available, it will be interesting to examine the nuances added to the controllability profiles of brain regions on the basis of the polarity of their connections.

It is important to note that we have taken an explicitly quantitative approach to controllability that differs from prior qualitative approaches. In important prior work, Liu and colleagues adopt a binary notion of controllability^55^ that is agnostic to the difficulty of the control task. In contrast, we ask how difficult the system is to control. In practice, these two questions can provide very different insights. Although a network may be generically controllable by any single node^55^,^56^, the actual control input may not be implementable because of actuator constraints and limitations^13^. A second important distinction between the two approaches is that structural controllability^56^ does not inform the design of realistic control algorithms. In contrast, we explore three controllability notions leading to the design of control strategies posited in the literature^13^ and ask how they relate to structural features of human brain anatomical networks. Our choice to focus on control strategies leads to a third important distinction between the two approaches. Namely, that the results presented in ref. 56 are generic in the sense that they hold for almost every choice of network parameters^57^ but they may fail to hold if certain symmetries or constraints are present (58, Section 15). In contrast, the three control strategies utilized here depend strongly on the properties of the network under study and are therefore sensitive to biologically relevant information.

Finally, we have focused on examining the controllability of single brain areas and reported salient relationships with cognitive control systems. However, future work may provide additional insights by studying controllability of sets of brain regions and their relationships to cognitive processes defined more broadly.

In conclusion, a fundamental understanding of the principles by which the brain transitions between diverse cognitive states enabling behaviour would necessarily have far-reaching implications for basic cognitive neuroscience and applications in myriad clinical domains^4^. Our results suggest that macroscale structural design could underlie basic cognitive control processes via the fundamental mechanism of network controllability. These findings lay the groundwork for future studies examining relationships between individual differences in network controllability diagnostics and behavioural, cognitive, clinical and genetic variables.

## Methods

### Human DSI data acquisition and preprocessing

DSIs were acquired for a total of eight subjects in triplicate (mean age 27±5 years, two female, two left handed) along with a *T*1-weighted anatomical scan at each scanning session^54^. DSI scans sampled 257 directions using a *Q*5 half-shell acquisition scheme with a maximum *b*-value of 5,000 and an isotropic voxel size of 2.4 mm. We utilized an axial acquisition with the following parameters: repetition time (TR)=11.4 s, echo time (TE)=138 ms, 51 slices, field of view (FoV) (231,231,123 mm). All participants volunteered with informed consent in accordance with the Institutional Review Board/Human Subjects Committee, University of California, Santa Barbara.

DSI data were reconstructed in DSI Studio (www.dsi-studio.labsolver.org) using *q*-space diffeomorphic reconstruction (QSDR)^53^. QSDR first reconstructs diffusion-weighted images in native space and computes the quantitative anisotropy (QA) in each voxel. These QA values are used to warp the brain to a template QA volume in Montreal Neurological Institute (MNI) space using the statistical parametric mapping (SPM) nonlinear registration algorithm. Once in MNI space, spin density functions were again reconstructed with a mean diffusion distance of 1.25 mm using three fibre orientations per voxel. Fibre tracking was performed in DSI studio with an angular cutoff of 55°, step size of 1.0 mm, minimum length of 10 mm, spin density function smoothing of 0.0, maximum length of 400 mm and a QA threshold determined by DWI signal in the colony-stimulating factor. Deterministic fibre tracking using a modified FACT algorithm was performed until 100,000 streamlines were reconstructed for each individual.

Anatomical scans were segmented using FreeSurfer^59^ and parcellated according to the Lausanne 2008 atlas included in the connectome mapping toolkit^12^. A parcellation scheme including 234 regions was registered to the B0 volume from each subject's DSI data. The B0 to MNI voxel mapping produced via QSDR was used to map region labels from native space to MNI coordinates. To extend region labels through the grey–white matter interface, the atlas was dilated by 4 mm. Dilation was accomplished by filling non-labelled voxels with the statistical mode of their neighbours' labels. In the event of a tie, one of the modes was arbitrarily selected. Each streamline was labelled according to its terminal region pair.

### Human DTI data acquisition and preprocessing

To complement the main analysis on DSI data acquired in triplicate from eight healthy human subjects, we also analysed DTI data from a separate set of 85 healthy human adult subjects^14^,^15^: mean age 34.96, s.d. 49.45; 3 female, 82 males; all right-handed. None of the subjects were colour blind. Informed written consent was obtained from each subject before the experimental sessions. All procedures were approved by the University of California, Santa Barbara Human Subjects Committee.

All scans were acquired at 3 T with a Siemens Tim Trio MRI scanner with a 12-channel-phased array head coil using an echo-planar diffusion-weighted technique acquired with iPAT and an acceleration factor of 2. The timing parameters of the pulse sequence were TE/TR=94/8,400 ms, 30 diffusion directions with a maximal *b*-value of 1,000 s mm^−2^ and two averages. Two *b*0 images were acquired. The matrix size was 128 × 128 and the slice number was 60. The field of view was 230 × 230 mm^2^ and the slice thickness 2 mm. Acquisition time was 9:08 min per DTI scan. In addition to diffusion scans, a three-dimensional (3D) high-resolution T1-weighted sagittal sequence image of the whole brain was obtained by a magnetization-prepared rapid acquisition gradient-echo sequence with the following parameters: TR=15.0 ms; TE=4.2 ms; flip angle=9 degrees, 3D acquisition, FOV=256 mm; slice thickness=0.89 mm, matrix=256 × 256.

Following prior work^14^,^15^,^60^, motion artefact and image distortions caused by eddy currents were corrected by using NIfTI Tools to open each DTI data set and perform an affine alignment (12 degrees of freedom) of each diffusion-weighted image to the *b*0 image via the FLIRT function in FMRIB software library (FSL). In the current study, we did not correct for echo planar imaging (EPI) distortions. In this Siemens scanner, the geometric distortion for diffusion imaging from EPI was found in prior tests to be less than 2 mm (that is, less than a single voxel) and mainly along the anterior posterior (phase-encoding) direction. Because the resolution of the diffusion images was larger than the magnitude of the distortion, no correction was required.

As with the DSI data, anatomical scans were segmented using FreeSurfer^59^ and parcellated according to the Lausanne 2008 atlas included in the connectome mapping toolkit^12^. A parcellation scheme including 234 regions was registered to the *b*0 volume from each subject's DTI data. Tractography was performed in DSI studio, and the number of streamlines connecting each pair of regions was used to weight the edge connecting those regions.

### Macaque tract tracing data

To address the question of whether the relationship between controllability diagnostics and network topology (as measured by weighted degree) was conserved in non-human primates, we used a data set drawn from CoCoMac (ref. 16) that delineated 2,402 projections between 95 cortical and subcortical areas^17^. These connectivity data were on the basis of three extensive neuroanatomical compilations that collectively cover large parts of the cerebral cortex. Although these data may be partially incomplete, particularly for connections of motor, auditory and somatosensory areas^17^, they represent an extensive effort in tract tracing, and therefore have been used extensively in studies of primate connectivity^17^. The CoCoMac database contains information on studies that report the source and target site of tracer injections, thereby specifying the specific presence or absence of anatomical projections between brain regions.

### Network control theory

Our understanding of natural systems is intimately related to our ability to control them. Network control theory is a branch of traditional control theory in engineering that addresses the question of how to control a system whose components are linked in a web of interconnections; here the term *control* indicates perturbing a system to reach a desired state. Answering this question requires (i) knowledge regarding the network connectivity linking system components and (ii) knowledge regarding how system components act, that is, their *dynamics*. In turn, the theory provides predictions regarding the system's function. Critically, in contrast to traditional graph theory that provides descriptive statistics of network structure, network control theory offers mechanistic predictors of network dynamics. The ability to probe mechanistic predictors of brain function is the key to move efforts in the human connectome towards an understanding of human cognition.

Mathematically speaking, we can study the controllability of a network system by defining a network represented by the graph 
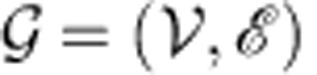
, where 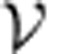 and 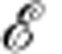 are the vertex and edge sets, respectively. Let *a*_*ij*_ be the weight associated with the edge 
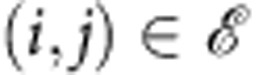
 and define the *weighted adjacency matrix* of 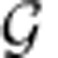 as *A*=[*a*_*ij*_], where *a*_*ij*_=0 whenever 
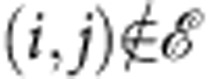
. Associate a real value (*state*) with each node, collect the nodes' states into a vector (*network state*) and define the map 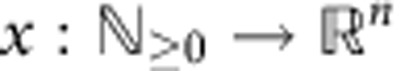 to describe the evolution (*network dynamics*) of the network state over time. Given the network and its dynamics, we can use network control theory to quantitatively examine how the network structure constrains the types of control that nodes can exert.

Here we are interested in applying the network control theory to the human brain. As a quintessentially complex biological system, the human brain offers several contexts in which to think about the notion of ‘control': both as a system that implements control and a system to be controlled. For example, control can be thought of as (i) the change in regional BOLD activity produced in response to neurofeedback in real-time fMRI, (ii) the change in regional neural activity elicited by external stimuli or (iii) the change in regional neural activity provoked by non-invasive brain stimulation. Each of these mechanisms initially alters the dynamics of single brain regions but can have consequences for the activity and function of distributed networks. Importantly, this notion of control is based on a very detailed mathematical construct and is therefore necessarily quite distinct from the cognitive neuroscientist's common notion of ‘cognitive control' and the distributed sets of brain regions implicated in its performance^2^. To minimize obfuscation, we henceforth refer to these two notions as ‘network control' and ‘cognitive control', respectively.

### Dynamic model of neural processes

To apply network control theory to the human brain, we must define a structural brain network and a model for the dynamics of neural processes. We define both based on prior work in human systems neuroscience. We define structural brain networks by subdividing the entire brain into anatomically distinct brain areas (network nodes), over five levels of spatial resolution from 83 regions to greater than 1,000 regions^61^. Consistent with prior work^14^,^15^,^60^, we connect nodes by the number of white matter streamlines identified by a commonly used deterministic tractography algorithm (for details on the tractography implementation, see ref. 54 and Supplementary Methods). This procedure results in sparse, weighted, undirected structural brain networks for each subject (*N*=8) and each scanning session (*n*=3). Properties of this network include high clustering, short path length and strong modularity (see Supplementary Methods), consistent with prior studies of similar network data^60^. The definition of structural brain networks on the basis of tractography data in humans follows from our primary hypothesis that control features of neural dynamics are in part determined by the structural organization of the brain's white matter tracts.

To define the dynamics of neural processes, we draw on prior models linking structural brain networks to resting state functional dynamics^44^. Although neural activity evolves through neural circuits as a collection of *nonlinear* dynamic processes, these prior studies have demonstrated that a significant amount of variance in neural dynamics as measured by fMRI can be predicted from simplified *linear* models. (See Methodological Considerations for additional discussion on the strengths and weaknesses of the linear model approach.) On the basis of this literature, we employ a simplified noise-free linear discrete-time and time-invariant network model:


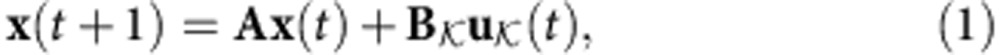


where 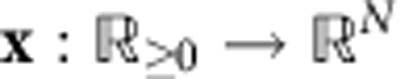 describes the state (that is, the magnitude of neurophysiological activity) of brain regions over time, and 
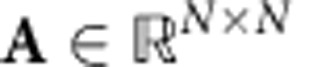
 is a symmetric and weighted adjacency matrix. In our case, we construct a weighted adjacency matrix whose elements indicate the number of white matter streamlines connecting two different brain regions—denoted here as *i* and *j*—and we stabilize this matrix by dividing by the the mean edge weight. While the model employed above is a discrete-time system, we find that the controllability Gramian is statistically similar to that obtained in a continuous-time setting (see Supplementary Methods).

More generally, we note that the network control theory framework is agnostic to the exact type of ‘activity' that the system produces. However, the model we write down above is a simplified ‘activity' dynamics that has previously been used to model both neural activity^45^ and regional BOLD activity^44^. In the context of our work in this paper, we use these dynamics to model fMRI BOLD magnitudes and their coherence across brain regions; however, future work may address the utility of this same construct in understanding different temporal scales of brain dynamics.

The diagonal elements of the matrix **A** satisfy *A*_*ii*_=0. The input matrix 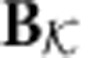 identifies the control points 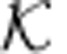 in the brain, where 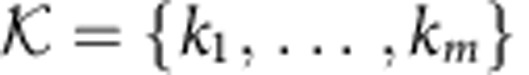 and


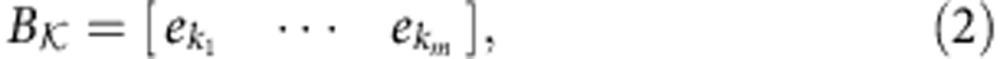


and *e*_*i*_ denotes the *i*-th canonical vector of dimension *N*. The input 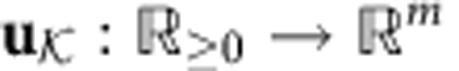 denotes the control strategy.

### Network controllability

To study the ability of a certain brain region to influence other regions in arbitrary ways, we adopt the control theoretic notion of *controllability*. Controllability of a dynamical system refers to the possibility of driving the state of a dynamical system to a specific target state by means of an external control input^55^. Classic results in the control theory ensure that controllability of the network (Equation 1) from the set of network nodes 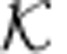 is equivalent to the controllability Gramian 
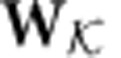
 being invertible, where


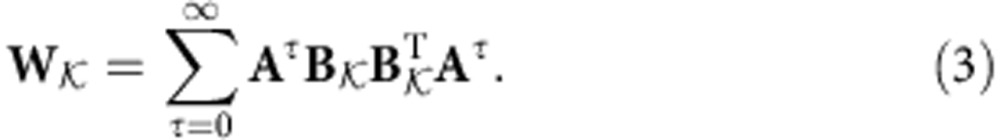


We utilize this framework to choose control nodes one at a time, and thus the input matrix *B* in fact reduces to a one-dimensional vector.

Besides ensuring controllability, the eigenvalues of the controllability Gramian are a quantitative measure of the magnitude of the control input that drives a network to a desired target state^62^, and the structure of the Gramian itself provides systematic guidelines for the selection of control areas that can theoretically optimize cognitive functions. While the magnitude of the control input may not be the unique feature to take into account when controlling brain dynamics^63^, it allows us to better understand the relationship between the structural organization of the brain and its dynamics, and opens the door to the development of novel diagnostics and opportunities for intervention. See Supplementary Note 1.

### Network controllability diagnostics

We examine three diagnostics of controllability utilized in the network control literature: *average controllability*, *modal controllability* and *boundary controllability*. See Supplementary Methods for additional details of these calculations.

### Average controllability

Average controllability of a network equals the average input energy from a set of control nodes and over all possible target states^64^,^65^. As a known result, average input energy is proportional to Trace
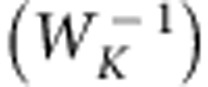
, the trace of the inverse of the controllability Gramian. Instead, we adopt Trace(*W*_*K*_) as a measure of average controllability for two main reasons: first, Trace
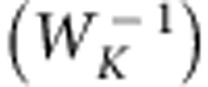
 and Trace(*W*_*K*_) satisfy a relation of inverse proportionality (see Supplementary Methods), so that the information obtained from the two metrices are correlated with one another and, second, *W*_*K*_ is typically very ill-conditioned (see paragraph ‘Global Controllability') even for coarse network resolutions, so that Trace
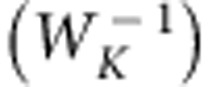
 cannot be accurately computed even for small brain networks. It should be noted that Trace(*W*_*K*_) encodes a well-defined control metric, namely the energy of the network impulse response or, equivalently, the network *H*_2_ norm^62^. Regions with high average controllability are, on average, most influential in the control of network dynamics over all different target states.

### Modal controllability

Modal controllability refers to the ability of a node to control each evolutionary mode of a dynamical network^66^, and can be used to identify states that are difficult to control from a set of control nodes. Modal controllability is computed from the eigenvector matrix *V*=[*v*_*ij*_] of the network adjacency matrix **A**. By extension from the PBH test^62^, if the entry *v*_*ij*_ is small, then the *j*-th mode is poorly controllable from node *i*. Following ref. 13, we define 
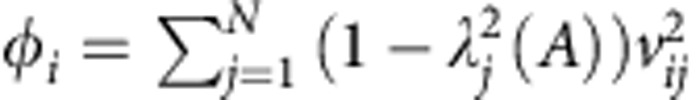
 as a scaled measure of the controllability of all *N* modes *λ*_1_(*A*),…,*λ*_*N*_(*A*) from the brain region *i*. Regions with high modal controllability are able to control all the dynamic modes of the network, and hence to drive the dynamics towards hard-to-reach configurations.

### Boundary controllability

Boundary controllability measures the ability of a set of control nodes to decouple the trajectories of disjoint brain regions. To evaluate the boundary controllability of different brain regions, we proceed as follows. First, we compute a *robust partition* of the brain network as described in ref. 67, and we identify the set of *N*_1_ boundary nodes. We assign to these boundary nodes the boundary controllability value of 1. Second, following ref. 13, we determine the two partitions of the least controllable subnetwork from its Fiedler eigenvector, and we identify the additional boundary nodes. We assign to these boundary nodes the boundary controllability value of (*N*−*N*_1_)/*N*. Finally, we iterate this process until all nodes have been assigned a boundary controllability value.

Average, modal and boundary controllability each provide a scalar value for each brain region. To enable direct comparison between controllability diagnostics and across different subjects, we perform ranking and normalization steps. In particular, for each of the controllability diagnostics we (i) rank the scalar values for each subject and (ii) average the ranked values across the subjects. Code is available on request.

## Additional information

**How to cite this article:** Gu, S. *et al*. Controllability of structural brain networks. *Nat. Commun.* 6:8414 doi: 10.1038/ncomms9414 (2015).

## Supplementary Material

Supplementary Information Supplementary Note 1, Supplementary Methods and Supplementary References

## Figures and Tables

**Figure 1:**
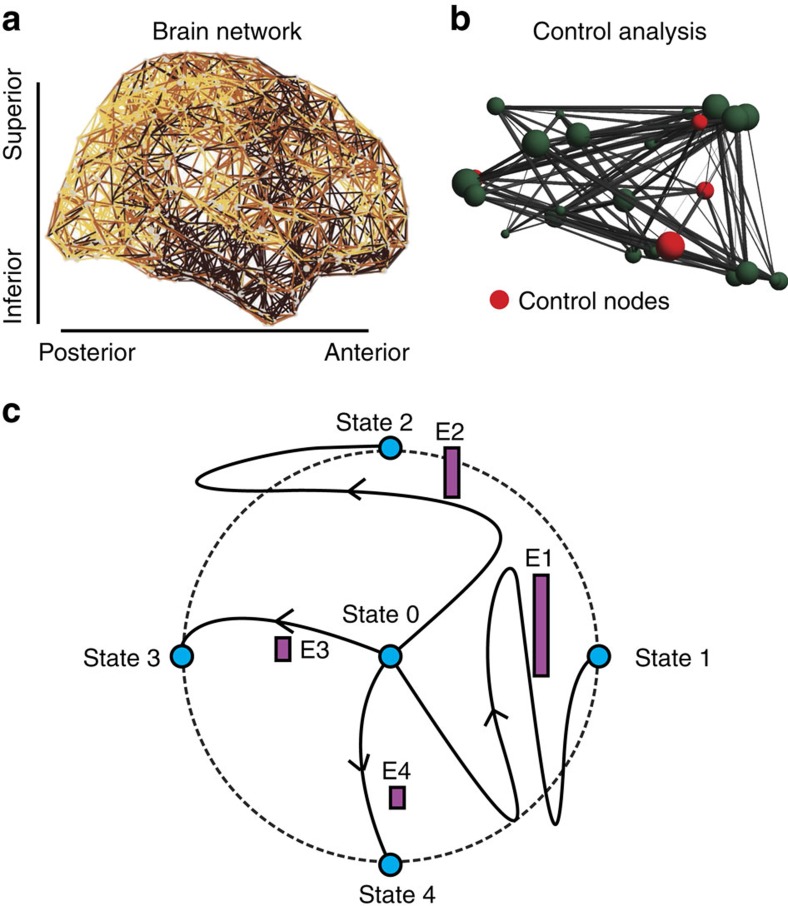
Conceptual schematic. From weighted brain networks (**a**), we estimate control points (**b**) whose large-scale regional activity can move the brain into new trajectories that traverse diverse cognitive functions (**c**). In **c**, we show the original state of the system (state 0), as well as four possible states (indicated by the blue circles) that are equidistant from state 0 in the state space (indicated by the black circular line), and which can be reached by trajectories that are more or less energetically costly (indicated by the height of the purple bars).
