# Supplementary material for: Controllability of structural brain networks
Source: Nat Commun. 2015 Oct 1;6:8414. doi: 10.1038/ncomms9414 (PMC4600713; doi:10.1038/ncomms9414)
Supplement: Supplementary Information — Supplementary Note 1, Supplementary Methods and Supplementary References [file ncomms9414-s1.pdf]

Supplementary Note 1

Interpretations Dependent on Model Assumptions Network controllability differs significantly from the static graph theoretical approaches that are increasingly used in studies of human brain connectivity. Network controllability models brain dynamics based on two features: (i) a structural connectivity matrix and (ii) an equation of state defining the dynamics that occur on top of that structure. The theoretical predictions of network controllability diagnostics are therefore dependent on the accuracy of these two features. Here we utilize state-of-the art DSI imaging techniques [1] and tractography reconstruction algorithms [2] to estimate white matter streamlines from the medial to lateral surfaces, and to distinguish their crossings [3]. An underlying assumption of this approach is that the number of streamlines is proportional to the strength of structural connectivity; this assumption has important exceptions but is most reasonable for cortico-cortical control, which is the primary area of investigation here (see SI for results from alternative weighting schemes). The equation of state that we utilize is based on extensive prior work demonstrating its utility in predicting resting state functional connectivity [4] and in providing similar brain dynamics to more complicated models [5]. Nevertheless, this model is simple, and our interpretations are dependent on its assumptions.

Considerations in the Use of Linear Models to Probe Nonlinear Neural Dynamics Controlling neural dynamics is a complex problem for many reasons. These include the facts that (i) neural systems exhibit nonlinear and stochastic dynamics, and (ii) neural systems arise from the intricate interconnection of a large number of components. Because the above two aspects of complexity are rather independent, in our work we focus on the complexity due to the interconnection structure of neural systems, and focus on linear dynamics only. This assumption of linear dynamics is commonly accepted in the study of network systems, and particularly when a graph-theoretic interpretation of certain dynamical properties is preferred; e.g., see [6–11]. Using this approach, we are able to identify important relationships between the interconnection structure (estimated from white matter tractography) and the evolution of neural dynamics at the level of the activity of large-scale brain regions from a control-theoretic perspective. These results are consistent with prior work providing evidence that the large-scale structural connectivity of neural systems is tightly tied to its dynamics as measured by fMRI [4,12]. In addition, these results are consistent with prior work providing evidence that structural connectivity is tightly tied to the brain's functionality as measured by cognitive scores and behavior: for example, properties of structural connectivity provide a prediction of intelligence [13], an understanding of cognitive disfunction in disease [14,15], a putative mechanism for Alzheimer's disease and fronto-temporal dementia [16], a correlate of inattention and hyperactivity in adolescent boys [17], a prediction of resting-state function [4,18,19], a genetic basis of memory deficits in Alzheimer's disease [20], and an understanding of working memory development in infants [21].

Role of Directionality in Brain Network Controllability The linear model of brain dynamics provided in Equation 1 in the main manuscript requires the definition of an adjacency matrix summarizing the structural connectivity between network nodes. We utilize a symmetric, undirected

adjacency matrix extracted from state-of-the-art diffusion spectrum imaging, a modality which does not establish directional flow between brain regions. Based on prior evidence from tract tracing studies in the macaque monkey [22], it is possible that white matter connections contain some unidirectional connections, in which either (i) region A projects to region B but region B does not project to region A, or (ii) region A projects to region B with a different strength than region B projects to region A. In the seminal work by Felleman and Van Essen (1991), it was estimated that unidirectional connections make up approximately 4% of the total number of connections. Thus, even in the case of structural connectivity in macaque monkeys, models which assume bidirectionality are arguably accurate to first order. In the case of the human, we do not have tract tracing data and instead rely on recent advances in diffusion imaging tractography which does not establish directionality, and therefore leads to a symmetric adjacency matrix. Nevertheless, should an imaging technique become available that would provide directional information between regions of interest, this information could be incorporated into the adjacency matrix used in Equation 1. Based on work in other control domains [23], the incorporation of directed structural connections may provide additional insight into control strategies available to or utilized by the human brain.

Implications of Weak Global Controllability The smallest eigenvalues of the controllability Gramian provide a mathematical measurement of how easy or difficult it is to move the system into **any** state, but do not provide any measurement of how easy or difficult it is to move the system into a **specific** state. It is possible for a system to be easy to move to the set of X states, but impossible to move to the set of Y states, and in this case the system would display some small eigenvalues (in fact of zero magnitude) of the controllability Gramian indicating that the system is difficult to control. The fact that there are states in which the human brain can move with ease (e.g., wakefulness, sleep, anesthesia, multiple task states, etc.) is fully consistent with low global controllability, and instead suggests that there are certain transitions between states that the brain has been optimized to perform with ease, potentially at the cost of making other transitions more difficult or impossible.

Supplementary Methods

Controllability of Network Systems Consider a network represented by the directed graph $\mathcal{G} = (\mathcal{V}, \mathcal{E})$, where $\mathcal{V}$ and $\mathcal{E}$ are the vertex and edge sets, respectively. Let a_{ij} be the weight associated with the edge $(i, j) \in \mathcal{E}$, and define the *weighted adjacency matrix* of $\mathcal{G}$ as $A = [a_{ij}]$, where $a_{ij} = 0$ whenever $(i, j) \notin \mathcal{E}$. We associate a real value (*state*) with each node, collect the nodes states into a vector (*network state*), and define the map $x : \mathbb{N}_{\geq 0} \to \mathbb{R}^n$ to describe the evolution (*network dynamics*) of the network state over time. In particular, the network dynamics are described by the discrete time, linear, and time-invariant recursion

$$x(t + 1) = Ax(t). \tag{1}$$

Let a subset of nodes $\mathcal{K} = \{k_1, \ldots, k_m\}$ be independently controlled, and let

$$B_{\mathcal{K}} := \begin{bmatrix} e_{k_1} & \cdots & e_{k_m} \end{bmatrix} \tag{2}$$

be the *input matrix*, where e_i denotes the i-th canonical vector of dimension n. The network with control nodes $\mathcal{K}$ reads as

$$x(t+1) = Ax(t) + B_{\mathcal{K}} u_{\mathcal{K}}(t), \tag{3}$$

where $u_{\mathcal{K}} : \mathbb{N}_{\geq 0} \to \mathbb{R}$ is the control signal injected into the network via the nodes $\mathcal{K}$. The network (3) is controllable in T steps by the nodes $\mathcal{K}$ if, for every state x_{f}, there exists a control input $u_{\mathcal{K}}$ such that $x(T) = x_{\mathrm{f}}$ with $x(0) = 0$ [24]. Controllability of dynamical systems can be ensured by different structural conditions [24,25]. For instance, let $\mathcal{C}_{\mathcal{K},T}$ be the *controllability matrix* defined as

$$\mathcal{C}_{\mathcal{K},T} := \begin{bmatrix} B_{\mathcal{K}} & AB_{\mathcal{K}} & \cdots & A^{T-1}B_{\mathcal{K}} \end{bmatrix}.$$

The network (3) is controllable in T steps by the nodes $\mathcal{K}$ if and only if $\mathcal{C}_{\mathcal{K},T}$ is of full row rank. A different condition for controllability with an infinite number of steps is presented in the main text.

In our context, the network (3) describes the evolution of a neural process of interest, where the network matrix A is constructed from diffusion spectrum imaging data, the state x contains the status of the brain regions throughout the process, and the control input $u_{\mathcal{K}}$ corresponds to the stimulations of the controlled regions $\mathcal{K}$. Our objective is to quantify the possibility and the difficulty to control the neural process between different states via external stimulation, and to identify the brain regions that are primarily involved in the control task.

Continuous and Discrete-Time Controllability Our controllability analysis can be applied to both continuous-time and discrete-time systems. In this paper, we opted for a discrete-time representation to emphasize the tight relationship and dependency between the interconnection structure and the system dynamics. But more generally, we believe that continuous-time and discrete-time representations are equally valid and applicable. In fact, we expect to obtain equivalent results for a continuous-time description such as $\dot{x} = Lx + Bu$, with $L = A - I$, and A our (normalized) adjacency matrix. In this case, L is a weighted Laplacian matrix. To test the validity of this expectation, we use the MATLAB function *lyap* to compute the controllability Gramian of stable continuous-time systems. We observe that the controllability Gramian of the continuous-time system is significantly correlated with the controllability Gramian of the discrete-time system, as measured by a Pearson correlation coefficient between the elements of the two matrices. The average Pearson correlation between continuous-time and discrete-time controllability Gramians is $r = 0.99$ (STD over subjects and scans 0.0015). These results indicate that we obtain equivalent results for continuous-time and discrete-time representations.

Correlation Between Degree and Average Controllability In the main manuscript, we describe a strong correlation between node degree and average controllability for the networks that we study. Here we provide a possible explanation for this effect. Due to the property $\mathrm{Trace}(\mathbf{ABC}) = \mathrm{Trace}(\mathbf{BCA})$, the average controllability with a single control node j equals the j-th diagonal elements of $(\mathbf{I} - \mathbf{A}^2)^{-1}$. Since A is stable, a first order approximation yields

$$(\mathbf{I} - \mathbf{A}^2)^{-1} \approx \mathbf{I} + \mathbf{A}^2, \tag{4}$$

and the j-th diagonal element of $(\mathbf{I}-\mathbf{A}^2)^{-1}$ is $1+\sum_{i=1}^N A_{ij}^2$. Since the degree of the j-th node equals $d_j=\sum_{i=1}^N A_{ij}$, a positive correlation between node degree and average controllability is mathematically expected in the networks that we study here.

Lower Bound on the Largest Eigenvalue of the Controllability Gramian In the main manuscript, we show that the smallest eigenvalue of the controllability Gramian is in fact much smaller than its largest counterpart. In fact, the largest eigenvalue of the controllability Gramian is lower bounded by 1. To see this, notice that

$$\lambda_{\max}(W_{\mathcal{K}})=\lambda_{\max}\left(\sum_{\tau=0}^{\infty}\mathbf{A}^\tau\mathbf{B}_{\mathcal{K}}\mathbf{B}_{\mathcal{K}}^{\mathsf{T}}\mathbf{A}^\tau\right)\geq\lambda_{\max}\left(\sum_{\tau=0}^{0}\mathbf{A}^\tau\mathbf{B}_{\mathcal{K}}\mathbf{B}_{\mathcal{K}}^{\mathsf{T}}\mathbf{A}^\tau\right)=\lambda_{\max}(\mathbf{B}_{\mathcal{K}}\mathbf{B}_{\mathcal{K}}^{\mathsf{T}})=1, \quad (5)$$

where the inequality follows from the fact that $\mathbf{A}^\tau\mathbf{B}_{\mathcal{K}}\mathbf{B}_{\mathcal{K}}^{\mathsf{T}}\mathbf{A}^\tau$ is positive semi-definite for all τ.

Additional Details for Control Methods Let the network be controllable in T steps, and let x_f be the desired final state in time T, with $||x_f||_2=1$. Define the energy of the control input $u_{\mathcal{K}}$ as

$$E(u_{\mathcal{K}},T)=||u_{\mathcal{K}}||_{2,T}^2=\sum_{\tau=0}^{T}||u_{\mathcal{K}}(\tau)||_2^2, \quad (6)$$

where T is the control horizon. The unique control input that steers the network state from $x(0)=0$ to $x(T)=x_f$ with minimum is [26]

$$u_{\mathcal{K}}^*(t)=B_{\mathcal{K}}^T(A^T)^{T-t-1}\mathcal{W}_{\mathcal{K},T}^{-1}x_f \quad (7)$$

with $t\in\{0,\ldots,T-1\}$. Then it can be seen that

$$E(u_{\mathcal{K}^*},T)=\sum_{\tau=0}^{T-1}||u_{\mathcal{K}}^*(\tau)||_2^2=x_f^T\mathcal{W}_{\mathcal{K},T}^{-1}x_f\leq\lambda_{\min}^{-1}(\mathcal{W}_{\mathcal{K},T}), \quad (8)$$

where equality is achieved whenever x_f is an eigenvector of $\mathcal{W}_{\mathcal{K},T}$ associated with $\lambda_{\min}(\mathcal{W}_{\mathcal{K},T})$. Because the control energy is limited in practical applications, controllable networks featuring small Gramian eigenvalues cannot be steered to certain states.

Average Controllability Average controllability of a network – formally defined as Trace$(\mathbf{W}_{\mathcal{K}}^{-1})$ – equals the average input energy from a set of control nodes and over all possible target states [27,28]. Motivated by the relation Trace$(\mathbf{W}_{\mathcal{K}}^{-1})\geq N^2/$Trace$(\mathbf{W}_{\mathcal{K}})$, recent results in the control of networked systems [29], and the fact that $\mathbf{W}_{\mathcal{K}}$ is close to singularity even for networks of small cardinality (see paragraph "Global Controllability" in the manuscript). It should be noticed that Trace(W_K) encodes a well-defined control metric, namely the energy of the network impulse response or, equivalently, the network H_2 norm [24]. Regions with high average controllability are most influential in the control of network dynamics over all different target states.

Modal Controllability The behavior of a dynamical system is fully determined by the eigenvalues (modes) and eigenvectors of its system matrix. Regarding controllability, the PBH test ensures that a system with matrix A is controllable by an input matrix B if and only if all its modes are controllable or, equivalently, if and only if there exists no left eigenvector of A orthogonal to the columns of B [26]. In particular, the ith mode is controllable by the matrix B if and only if $w_i B \neq 0$, where w_i is a left eigenvector of A associated with its ith mode.

In our setting, because the network matrix A is symmetric and the input matrix B is a collection of canonical vectors, the PBH test implies that the ith mode of the network A is not controllable by the ith node if and only if the ith entry of the ith eigenvector is zero. Let $V = [v_{ij}]$ be the matrix of eigenvectors of A. By extension from the PBH test, if the entry v_{ij} is small, than the jth mode is poorly controllable by node i. Following [30], we define $\phi_i = \sum_{j=1}^{N}(1 - \lambda_j^2(A))v_{ij}^2$ as a scaled measure of the controllability of all N modes $\lambda_1(A), \ldots, \lambda_N(A)$ from the brain region i. Regions with high modal controllability are able to control all the dynamic modes of the network, and hence to drive the dynamics towards hard-to-reach configurations. We note that our method to find influential "modal control" nodes is exact (non-heuristic) with respect to our definition [23].

Boundary Controllability In the main manuscript, we briefly describe our method for detecting boundary control points. This method is largely based on the algorithm proposed in [30]. However, for the application to brain networks derived from diffusion tractography, we have made two important modifications to more accurately estimate the initial partition and constrain the boundary point criteria as described in detail below.

Initial Partition The first modification concerns the definitions of the first level subnetworks for which we compute a two-partition based on the Fiedler eigenvector. In initial work, Pasqualetti et al. [30] suggest computing the Fiedler eigenvector of the adjacency matrix to create first level subnetworks defined by a two-partition. In contrast, we define this first level of subnetworks as composed of network communities, identified by maximizing the modularity quality function [31] using a Louvain-like [32] locally greedy algorithm [33]. Our choice is based on extensive recent literature demonstrating that the brain is composed of many subnetworks (not just 2) [34,35], which can be extracted using modularity maximization approaches [36–38], and which correspond to sets of brain areas performing related functions [37,39].

The modularity quality function provides an estimate of the quality of a hard partition of the $N \times N$ adjacency matrix $\mathbf{A}$ into network communities (whereby each brain region is assigned to exactly one network community) [31,40–43]

$$Q_0 = \sum_{ij}[A_{ij} - \gamma P_{ij}]\delta(g_i, g_j)\,, \tag{9}$$

where brain region i is assigned to community g_i, brain region j is assigned to community g_j, $\delta(g_i, g_j) = 1$ if $g_i = g_j$ and it equals 0 otherwise, γ is a structural resolution parameter, and P_{ij}

is the expected weight of the edge connecting node i and node j under a specified null model. Maximization of Q_0 yields a hard partition of a network into communities such that the total edge weight inside of communities is as large as possible (relative to the null model and subject to the limitations of the employed computational heuristics, as optimizing Q_0 is NP-hard [42–44]).

Because the modularity quality function has many near-degeneracies, it is important to perform the optimization algorithm multiple times [45]. We perform 100 optimizations of the Louvain-like locally greedy algorithm [33] for each adjacency matrix corresponding to a single scan. To distill a single representative partition, we create a consensus partition from these 100 optimizations based on statistical comparison to an appropriate null model [35].

In a final consideration, we choose a value for the structural resolution parameter γ. The choice $\gamma = 1$ is very common, but it is important to consider multiple values of γ to examine community structure at multiple scales [42,46,47]. Indeed, recent work has demonstrated that in some networks, a structural resolution parameter value that accurately captures the underlying community structure can be identified by the γ value at which the 100 optimizations produce similar partitions [35]. To quantitatively estimate similarity in partitions, we adopt the z-score of the Rand coefficient [48]. For each pair of partitions α and β, we calculate the Rand z-score in terms of the total number of pairs of nodes in the network M, the number of pairs M_α that are in the same community in partition α, the number of pairs M_β that are in the same community in partition β, and the number of pairs of nodes $w_{\alpha\beta}$ that are assigned to the same community both in partition α and in partition β. The z-score of the Rand coefficient comparing these two partitions is

$$z_{\alpha\beta} = \frac{1}{\sigma_{w_{\alpha\beta}}} w_{\alpha\beta} - \frac{M_\alpha M_\beta}{M}\,, \tag{10}$$

where $\sigma_{w_{\alpha\beta}}$ is the standard deviation of $w_{\alpha\beta}$. Let the *mean partition similarity* denote the mean value of $z_{\alpha\beta}$ over all possible partition pairs for $\alpha \neq \beta$. Let the *variance of the partition similarity* denote the variance of $z_{\alpha\beta}$ over all possible partition pairs for $\alpha \neq \beta$.

Empirically, we calculated a group adjacency matrix by averaging the adjacency matrices of all subjects and scans. We optimized the modularity quality function 100 times and we computed the mean and variance of the partition similarity for a range of γ values and for all 5 spatial resolutions. Across all atlases, we observed that the mean partition similarity was high and the variance of the partition similarity was low for values of γ ranging between 1.5 and 2. For Scale 125 (the atlas for which we report results in the main manuscript), we observed a maximum mean partition similarity and a minimum variance of partition similarity at $\gamma = 1.6$. We therefore chose to set $\gamma = 1.6$ for the remainder of the analysis in this study.

Boundary Point Criteria The second modification concerns the definition of a boundary point. After calculating the Fiedler eigenvector of a subnetwork to determine a partition of the subnetwork into two communities, we must identify "boundary points", which are nodes that contain connections to both communities. In the original work by Pasqualetti and colleagues, it was

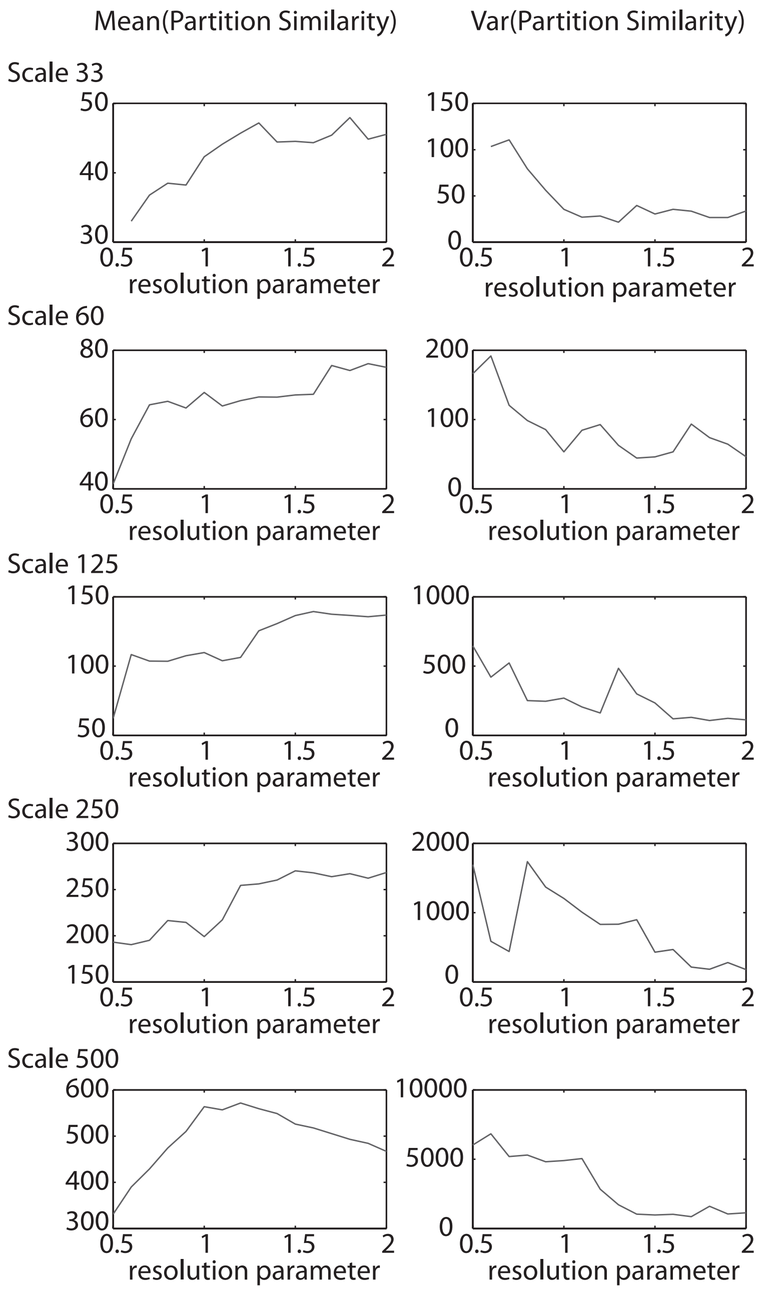

Supplementary Figure 1: **Partition Similarity As a Function of the Resolution Parameter** Mean *(left)* and variance *(right)* of the partition similarity estimated using the z-score of the Rand coefficient as a function of the structural resolution parameter γ, varies from 0.5 to 2 in increments of 0.1, for the 5 spatial scales of the Lausanne atlas [49] *(rows)*.

suggested that a boundary point was a node with any number of connections to both communities. However, in weighted brain networks we suggest that a more stringent definition is more appropriate for the following reason: practically all nodes in the brain have non-zero weighted connections to both identified communities. Therefore, we instead set a threshold ratio ρ to identify boundary points. Considering the adaptivity to the local measure, we set a threshold ratio ρ instead of a global threshold value. In detail, for a network $G = (V, E)$ with partition $P = (V_1, \cdots, V_n)$, a node $i \in V_k$ is called a boundary node if

$$\sum_{l \neq k} a_{kl} \geq \rho \cdot \max(A) \tag{11}$$

where A is the adjacency matrix. Here, $\max(A)$ can be replaced with other statistics and ρ needs to be chosen carefully. If ρ is too small, there will be no effect and the algorithm tends to add the total subnetwork as the set of boundary points. If ρ is too large, there will be only a few points recognized as the boundary points.

In the results described in the main manuscript, we set the threshold ratio to $\rho = 0.2$. To determine whether our results are robust to this choice, we calculate boundary controllability values across all regions in the Scale 125 atlas, for each scan using ρ values that vary between 0.15 and 0.25 in increments of 0.01. We then asked how similar regional control values were for different choices of ρ. Specifically, for any pair of ρ values, we computed the Pearson correlation coefficient between the vectors of regional control values for the two ρ values. We show the results of this analysis in Fig. 2. We observe that the boundary control values are highly similar across choices of ρ (minimum Pearson correlation approximately 0.68, corresponding to a $p = 0$, indicating that our results are robust to small variation in the boundary point criteria threshold.

Final Algorithm Thus, the final algorithm used in the calculation of boundary controllability in this paper can be summarized as follows. We begin with the application of a community detection method to the brain network to extract a partition of brain regions into network communities. We then recursively apply a Fiedler bipartition to add boundary nodes within communities, with the goal of improving the local controllability of the network. At each stage of the algorithm, we define the boundary nodes of the network as the nodes that maintain edges to nodes in other

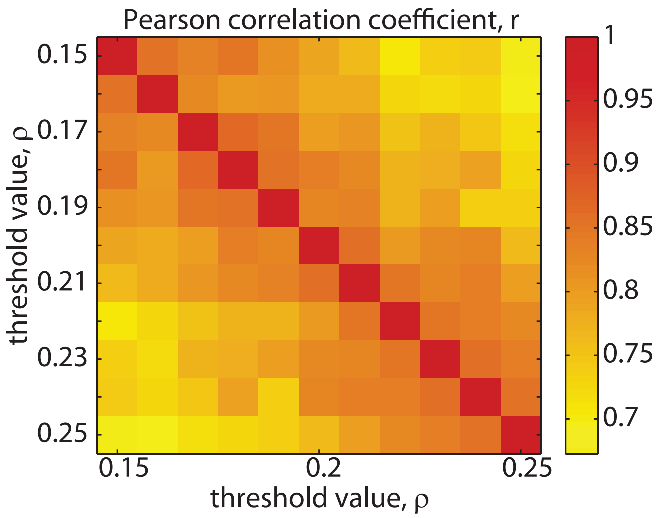

Supplementary Figure 2: **Effect of Boundary Point Criteria Threshold** Color indicates Pearson correlation coefficient, r, between the vectors of boundary controllability values estimated for pairs of ρ values in the range $0.15 - 0.25$ in increments of 0.01.

communities. Algorithmically, we can write:

Algorithm 1: Algorithm for the Selection of Boundary Control Nodes

---

Data: Network $G = (V, E)$ with adjacency matrix $A = (a_{ij})$, Number of control nodes m, threshold ratio ρ;

Result: Control Nodes Index Set $\mathcal{K}$;

Define an empty set of control nodes $\mathcal{K} = \emptyset$;
Initialize the partition $\mathcal{P}$ with the result of a community detection algorithm and initialize the boundary nodes set $\mathbf{B} = \emptyset$;
Add the boundary points of the initial partition;
while $|\mathcal{K}| < m$ **do**
  Select least controllable community $l = \arg\min\{\lambda_{min}(W_{i,\infty}), i = 1, ..., |\mathcal{P}|\}$;
  Compute Fiedler two-partition P_f of l-th community;
  Compute boundary nodes B_f of P_f with the given threshold ratio ρ;
  Update partition $\mathcal{P}$ with P_f;
  Update control nodes with boundary nodes $\mathcal{K} = \mathcal{K} \cup B_f$;
end
return $\mathcal{K}$.

Association of Brain Regions to Cognitive Systems To examine the relationship between controllability diagnostics and cognitive systems, we developed a map of brain areas to a set of cognitive systems previously defined in the literature: the fronto-parietal, cingulo-opercular, dorsal attention, ventral attention, default mode, motor and somatosensory, auditory, visual, subcortical

systems [39]. Such a mapping was inspired by a recent paper from Power et al. (2012) who associated 264 brain areas to these cognitive systems, defined by a clustering technique applied to functional brain networks [39]. Similar to previous work [39], our association of areas to systems is a gross approximation and it should not be interpreted as indicating that areas have single functions. We use this association only as a pragmatic means to assess whether controllability diagnostics are differentially identified in distributed neural circuits.

The 234 areas examined in the main manuscript were drawn from 42 cortical structures. Here we associate these 42 structures to the set of 9 cognitive systems:

- **Lateral Orbitofrontal** In the Power et al. (2012) decomposition, portions of lateral orbitofrontal cortex (or BA 47) are assigned to default mode, salience, and ventral attention systems. To choose a single association for this region, we turned to the wider literature. In a recent meta-analysis, Zald and colleagues examined the role of medial and lateral orbitofrontal cortex in widespread functional networks [50]. The lateral orbitofrontal cortex showed co-activations with prefrontal regions and areas involved in cognitive functions including language and memory but not with areas of the default mode, autonomic, and limbic systems. Rothkirch et al. (2012) similarly demonstrated that lateral orbitofrontal cortex appears to be modulated by implicit motivational value, rather than salience [51], arguing against its inclusion in the salience system. Anderson and colleagues suggest that lateral orbitofrontal cortex provides a specificity in top-down control of attention in collaboration with dorsolateral prefrontal cortex [52]. Cognitive system assignment: "Ventral Attention".
- **Pars Orbitalis** In the Power et al. (2012) decomposition, portions of pars orbitalis (or BA 47) are assigned to default mode, salience, and ventral attention systems. To choose a single association for this region, we turned to the wider literature. The pars orbitalis is a part of the ventrolateral prefrontal cortex, and is known to play a role in cognitive control processes [53], particularly in conflict adaptation [54], inhibition [55], which differ significantly from those enabled by the fronto-parietal network [56]. Cognitive system assignment: "Cingulo-Opercular".
- **Frontal Pole** In this parcellation scheme, the frontal pole corresponds to portions of BA 9 and 10. These areas form hubs of the fronto-parietal cognitive control system [57]. Cognitive system assignment: "Fronto-parietal".
- **Medial Orbitofrontal**. The medial frontal cortex is one of the key hubs of the fronto-parietal network [57,58]. Cognitive system assignment: "Fronto-parietal".
- **Pars Triangularis** In this parcellation scheme, the pars triangularis corresponds to portions of BA 45, and therefore maps to the fronto-parietal cognitive control system [56]. Cognitive system assignment: "Fronto-parietal".
- **Pars Opercularis** The pars of opercularis (corresponding roughly to BA 44) forms a hub of the cingulo-opercular cognitive control system [56]. Cognitive system assignment: "Cingulo-Opercular".

- **Rostral Middle Frontal** The rostral middle frontal cortex, corresponding roughly to BA 10, forms a hub of the cingulo-opercular cognitive control system [56]. Cognitive system assignment: “Cingulo-Opercular”.
- **Superior Frontal**. In the Power et al. (2012) decomposition, portions of the superior frontal cortex are predominantly affiliated with the default mode system, consistent with previous literature [59–62]. Cognitive system assignment: “Default Mode”.
- **Caudal Middle Frontal** The caudal middle frontal cortex is a prefrontal cortical structure broadly associated with executive function [63,64], top-down control [65], and secondary motor processes [66,67]. Cognitive system assignment: “Fronto-parietal”.
- **Precentral** The precentral cortex is part of the somatosensory system. Cognitive system assignment: “Somatosensory”.
- **Paracentral** The paracentral cortex is part of the somatosensory system. Cognitive system assignment: “Somatosensory”.
- **Rostral Anterior Cingulate** The anterior cingulate is a hub of the cingulo-opercular network [68–73]. Cognitive system assignment: “Cingulo-Opercular”.
- **Caudal Anterior Cingulate** The anterior cingulate is a hub of the cingulo-opercular network [68–73]. Cognitive system assignment: “Cingulo-Opercular”.
- **Posterior Cingulate**. The posterior cingulate is a known hub of the default mode system [58,74,75]. Cognitive system assignment: “Default Mode”.
- **Isthmus Cingulate** The isthmus cingulate is thought to be a hub of the default mode system [76] and of the limbic system [77]. Cognitive system assignment: “Default Mode”.
- **Post Central** The postcentral cortex is part of the somatosensory system. Cognitive system assignment: “Somatosensory”.
- **Supramarginal** The supramarginal gyrus appears to play a role in the dorsal [78] and ventral [79] attention networks, and executive function more broadly [80,81]. In the Power et al. (2012) decomposition, this area was assigned to the cingulo-opercular system [39]. Cognitive system assignment: “Cingulo-Opercular”.
- **Superior Parietal** The superior parietal cortex plays a role in both the dorsal attention system [82,83] and the somatosensory-motor system [84]. Cognitive system assignment: “Dorsal Attention”.
- **Inferior Parietal**. The inferior parietal cortex is one of the key hubs of the fronto-parietal network [57,58]. Cognitive system assignment: “Fronto-parietal”.
- **Precuneus** The precuneus is a hub of the default mode system [85,86]. Cognitive system assignment: “Default Mode”.

- **Cuneus** The cuneus is a part of the visual system [82,87,88]. Cognitive system assignment: “Visual”.
- **Pericalcarine** The pericalcarine is a part of the visual system [87,89]. Cognitive system assignment: “Visual”.
- **Lateral Occipital** The lateral occipital cortex is a part of the visual system [90]. Cognitive system assignment: “Visual”.
- **Lingual** The lingual gyrus is a part of the visual system [87,91]. Cognitive system assignment: “Visual”.
- **Fusiform** The lingual gyrus is a part of the visual system [82]. Cognitive system assignment: “Visual”.
- **Parahippocampal** The parahippocampal cortex has been associated with many cognitive processes including visuospatial processing and episodic memory [92]. Cognitive system assignment: “Other”.
- **Entorhinal cortex** The entorhinal cortex encodes visual information [93]. Cognitive system assignment: “Visual”.
- **Temporal Pole** The temporal pole plays a role in language processing, including naming [94], and in social and emotional processing [95]. Cognitive system assignment: “Other”.
- **Inferior Temporal** The inferior temporal cortex is associated with visual processing [96], emotion perception of visual objects [97], and shape recognition [98]. Cognitive system assignment: “Visual”.
- **Middle Temporal** The middle temporal cortex is associated with cognitive control processes [99], theory of mind [100], and social cognition [101]. Cognitive system assignment: “Other”.
- **Bank of the Superior Temporal Sulcus** The bank of the superior temporal sulcus forms a part of the early cortical auditory network [102]. Cognitive system assignment: “Auditory”.
- **Superior Temporal** The superior temporal cortex forms a part of the auditory system [103]. Cognitive system assignment: “Auditory”.
- **Transverse Temporal** The transverse temporal cortex forms a part of the auditory system [104]. Cognitive system assignment: “Auditory”.
- **Insula**. The insula is one of the key hubs of the fronto-parietal network [57,58]. Cognitive system assignment: “Fronto-parietal”.
- **Thalamus**. Cognitive system assignment: “Subcortical”.
- **Caudate**. Cognitive system assignment: “Subcortical”.

- **Putamen**. Cognitive system assignment: “Subcortical”.
- **Pallidum**. Cognitive system assignment: “Subcortical”.
- **Nucleus Accumbens**. Cognitive system assignment: “Subcortical”.
- **Hippocampus**. Cognitive system assignment: “Subcortical”.
- **Amygdala**. Cognitive system assignment: “Subcortical”.
- **Brainstem**. Cognitive system assignment: “Other”.

Methodological Considerations and Checks In the main manuscript, we utilize a parcellation of the cortical and subcortical tissue into $N = 234$ different brain regions. This parcellation is in fact part of a wider family of Lausanne atlas parcellations that include the following:

- Scale 33: $N = 83$ brain regions
- Scale 60: $N = 129$ brain regions
- Scale 125: $N = 234$ brain regions
- Scale 250: $N = 463$ brain regions
- Scale 500: $N = 1015$ brain regions

This multi-scale atlas has previously been used to examine the hierarchical nature of brain network topography [49]. In this and the following sections of this supplement, we examine the reproducibility of our results obtained using Scale 125 (described in the main manuscript) across the remaining spatial resolutions provided by the other 4 atlases.

Global Controllability Across Spatial Scales We calculated the global controllability of each node in each atlas for each person and scan. We observed that the mean global controllability (averaged across subjects, scans, and nodes) decreases with the spatial scale of the atlas: see Table1. Note: here we report the mean and STD of global controllability diagnostics over brain regions.

Supplementary Table 1: Global Controllability Diagnostic Values (GC) over the 5 Scales of the Lausanne Atlas Family.

Scale	Number of Nodes	mean GC	STD GC
33	83	2.55×10^{-21}	1.61×10^{-21}
60	129	5.78×10^{-22}	3.88×10^{-22}
125	234	4.52×10^{-23}	3.59×10^{-23}
250	463	7.10×10^{-25}	7.65×10^{-25}
500	1015	2.09×10^{-27}	7.23×10^{-27}

Reproducibility of Controllability Diagnostics Across Spatial Scales In the main manuscript, we show the anatomical distribution of the 3 controllability diagnostics over the $N = 234$ brain regions of the Scale 125 atlas. In Fig. 3 of this supplement, we show that the anatomical distribution of average controllability is visually similar across all 5 spatial scales assessed with the entire Lausanne atlas family. In Fig. 4 and Fig. 5, we show a similar reproducibility of the anatomical distribution of modal and boundary controllability, respectively.

Reproducibility of Degree-Controllability Correlations Across Spatial Scales In the main manuscript, we observed that for Scale 125 ($N = 234$) the degree was strongly positively correlated with the average controllability, strongly negatively correlated with the modal controllability, and neither strongly positively nor strongly negatively correlated with the boundary controllability. In Table 2, we report the correlations between degree and the 3 controllability diagnostics as a function of spatial resolution: from Scale 33 ($N = 83$) to Scale 500 ($N = 1015$). We observe that the degree-controllability correlations reported for Scale 125 are reproducibly observed across the remaining 4 spatial scales, comprising both higher and lower spatial resolutions.

Test-Retest Reliability of Controllability Diagnostics When proposing a new diagnostic of brain network architecture, it is critical to determine the reliability of those diagnostic values across iterative measurement. Here we capitalize on the fact that the same 8 subjects whose data are reported in the main manuscript were imaged over 3 different days. We utilize these iterative scans to assess the test-retest reliability of the 3 controllability diagnostics.

To compare the results among different scans and subjects, we consider the average correlation. Suppose we have n subjects and for each of them we have K scans with corresponding controllability values $c_1^i, \cdots, c_K^i$. The averaged correlation between controllability diagnostic values for subject i and subject j is defined as

$$R_{ij}^B = \frac{\sum_{s=1}^K \sum_{t=1}^K \operatorname{corr}(c_s^i, c_t^j)}{K^2} \tag{12}$$

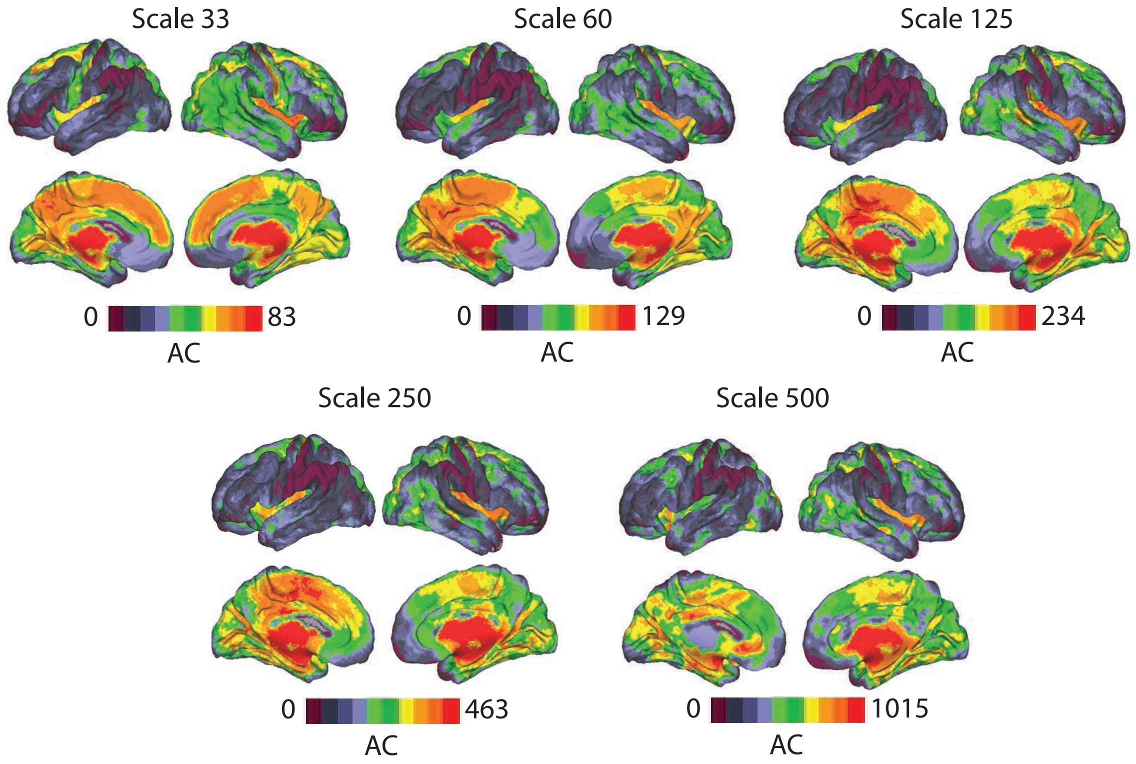

Supplementary Figure 3: **Average Controllability Across Spatial Scales** Surface visualizations of the ranked average controllability (AC) values over the 5 spatial scales of the Lausanne atlas [49].

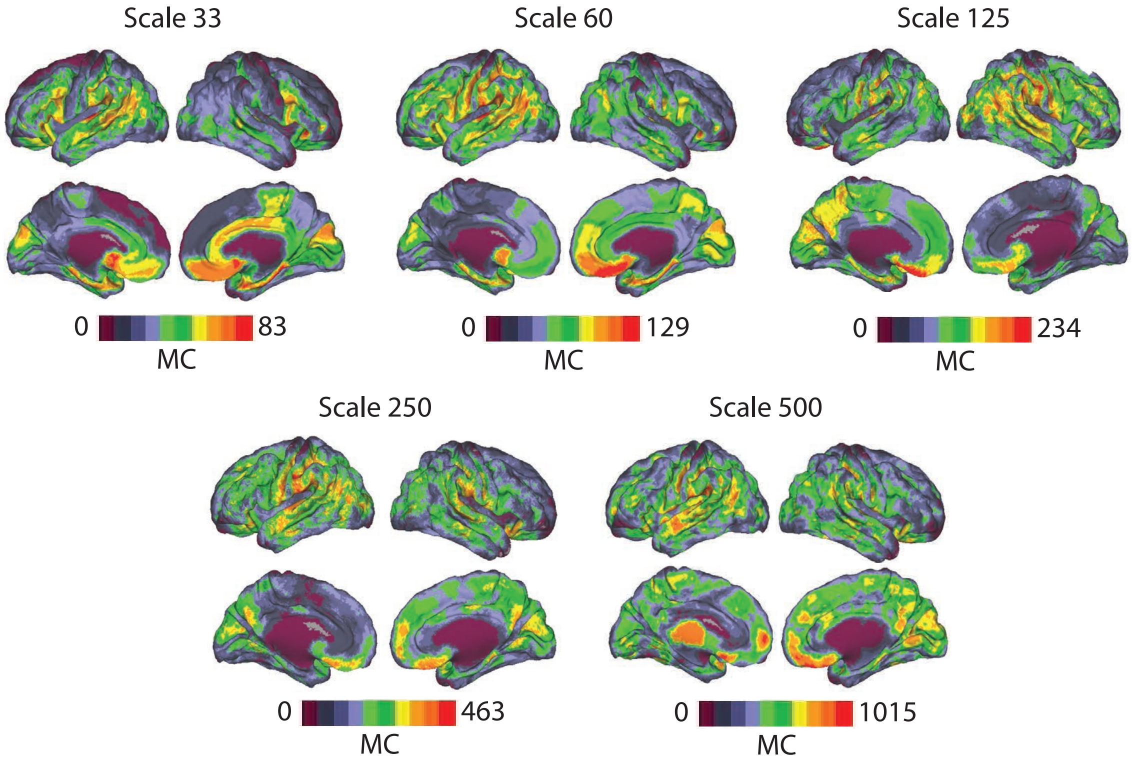

Supplementary Figure 4: **Modal Controllability Across Spatial Scales** Surface visualizations of the ranked modal controllability (MC) values over the 5 spatial scales of the Lausanne atlas [49].

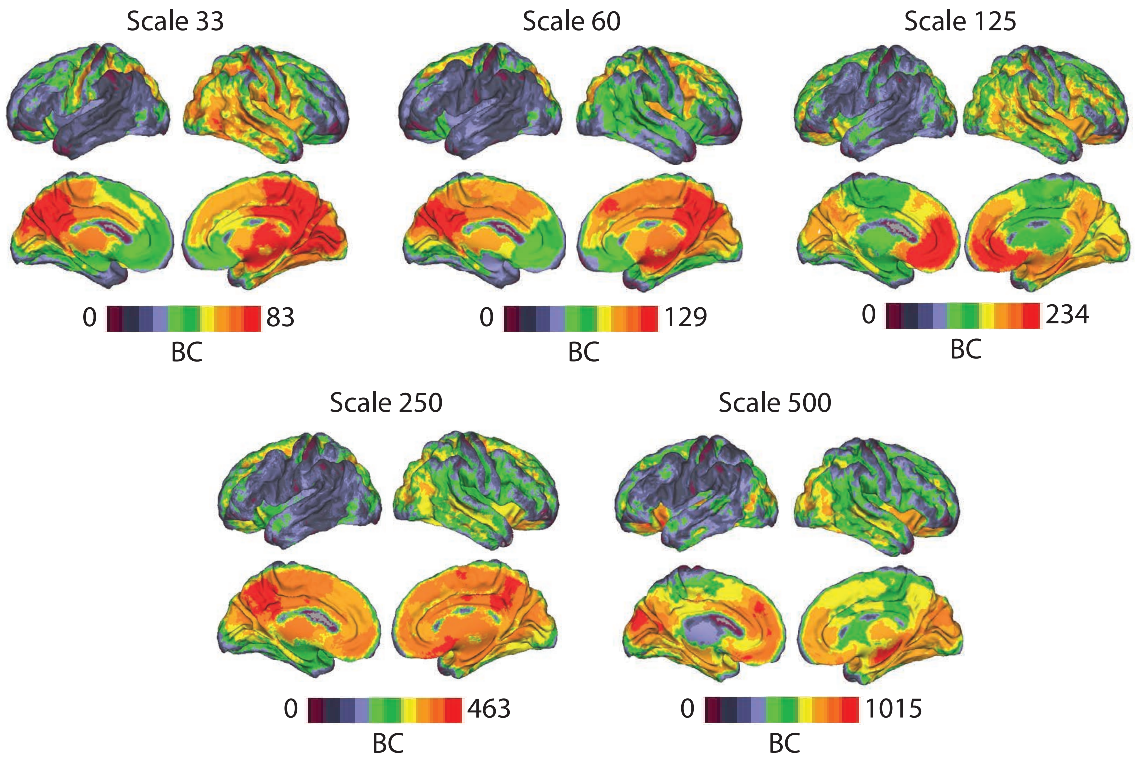

Supplementary Figure 5: **Boundary Controllability Across Spatial Scales** Surface visualizations of the ranked boundary controllability (BC) values over the 5 spatial scales of the Lausanne atlas [49].

Supplementary Table 2: Pearson correlation coefficients r between rank degree, average controllability (AC), boundary controllability (BC), and modal controllability (MC).

		Degree	AC	BC	MC
**Scale 33**					
	Degree	1.0000	0.9764	0.5225	-0.9923
	AC	0.9764	1.0000	0.6302	-0.9688
	BC	0.5225	0.6302	1.0000	-0.5120
	MC	-0.9923	-0.9688	-0.5120	1.0000
**Scale 60**					
	Degree	1.0000	0.9429	0.4733	-0.9912
	AC	0.9429	1.0000	0.6262	-0.9320
	BC	0.4733	0.6252	1.0000	-0.4806
	MC	-0.9912	-0.9320	-0.4806	1.0000
**Scale 125**					
	Degree	1.0000	0.9205	0.1385	-0.9937
	AC	0.9205	1.0000	0.1461	-0.9125
	BC	0.1385	0.1461	1.0000	-0.1270
	MC	-0.9937	-0.9125	-0.1270	1.0000
**Scale 250**					
	Degree	1.0000	0.9114	0.3310	-0.8626
	AC	0.9114	1.0000	0.4785	-0.7822
	BC	0.3310	0.4785	1.0000	-0.2968
	MC	-0.8626	-0.7822	-0.2968	1.0000
**Scale 500**					
	Degree	1.0000	0.9122	0.2709	-0.9962
	AC	0.9122	1.0000	0.3637	-0.9042
	BC	0.2709	0.3637	1.0000	-0.2366
	MC	-0.9962	-0.9042	-0.2366	1.0000

for subject $i \neq j$ and where s and t index scanning sessions, and corr indicates the calculation of a Pearson correlation coefficient. The average correlation between controllability diagnostic values for the same subject across scanning sessions is defined as

$$R^W_{ii} = \frac{\sum_{s \neq t} \mathrm{corr}(c^i_s, c^i_t)}{K(K-1)} \tag{13}$$

for $i = j$. We refer to the quantity R^B_{ij} as the average between-subject correlation and to the quantity R^W_{ii} as the average within-subject correlation.

We report the within- and between-subject correlations for all 3 controllability diagnostics and for global controllability across all 5 spatial scales of the Lausanne atlas family in Tab. 3. We observe that all 3 controllability diagnostics display significantly greater within-subject correlation than between-subject correlation, indicating that these diagnostics are statistically reproducible across scanning sessions and significantly different across individuals. The average and modal controllability display a relatively high mean R (approximately 0.90) and relatively low standard error. While still statistically reproducible across scanning sessions, the boundary controllability displays a lower mean R than the average and modal controllability, and a higher standard error. The global controllability is not reproducible across scanning sessions. These observations are consistently observed across the 5 spatial scales of the Lausanne atlas family of parcellations.

Reproducibility of Control Roles of Cognitive Systems In the main text, we observed that 30% of average control hubs lie in the default mode system, 32% of modal control hubs lie in the fronto-parietal and cingulo-opercular cognitive control systems, and 34% of boundary control hubs lie in the ventral and dorsal attention systems. Here we demonstrate that these results are qualitatively reproduced for different definitions of control hubs: namely, the 25 nodes with the highest control values (out of a possible 234 nodes), the 30 nodes with the highest control values (as shown in the main manuscript), or the 35 nodes with the highest control values. When control hubs are defined as the top 25 nodes, we observe that 32% of average control hubs lie in the default mode system, 33% of modal control hubs lie in the fronto-parietal and cingulo-opercular systems, and 33% of boundary control hubs lie in the ventral and dorsal attentional systems. When control hubs are defined as the top 35 nodes, we observe that 28% of average control hubs lie in the default mode system, 31% of modal control hubs lie in the fronto-parietal and cingulo-opercular systems, and 32% of boundary control hubs lie in the ventral and dorsal attentional systems. These results demonstrate that the presence of a controllability-by-system interaction is robust to small variation in the choice of the size of the control hub set.

Reproducibility of Differential Recruitment of Cognitive Systems to Network Control In the main text, we observed the presence of a controllability-by-system interaction, and interpreted this as indicative of the possibility that certain types of controllability may be utilized or enabled

Supplementary Table 3: Test-Retest Reliability of Controllability Diagnostics: average controllability (AC), boundary controllability (BC), modal controllability (MC) and global controllability (GC).

		AC	BC	MC	GC
**Scale 33**					
	Mean Within	0.9642	0.7250	0.9708	0.0674
	Mean Between	0.8966	0.3436	0.9191	0.0501
	STE Within	0.0222	0.1279	0.0119	0.0527
	STE Between	0.0227	0.2014	0.0168	0.0426
	p-value	5.7e-11	2.5e-6	9.2e-12	0.4626
**Scale 60**					
	Mean Within	0.9510	0.6146	0.9519	0.0662
	Mean Between	0.8449	0.3465	0.8544	0.0501
	STE Within	0.0283	0.1552	0.0199	0.0590
	STE Between	0.0333	0.1351	0.0261	0.0387
	p-value	4.0e-12	2.8e-6	9.5e-15	0.3076
**Scale 125**					
	Mean Within	0.9404	0.5147	0.9348	0.0782
	Mean Between	0.8036	0.1954	0.7900	0.0527
	STE Within	0.0298	0.1311	0.0234	0.0508
	STE Between	0.0383	0.1638	0.0350	0.0449
	p-value	5.3×10^{-14}	1.8×10^{-6}	1.1×10^{-16}	0.1442
**Scale 250**					
	Mean Within	0.9320	0.5192	0.9230	0.0481
	Mean Between	0.7751	0.2208	0.7451	0.0391
	STE Within	0.0256	0.2012	0.0227	0.0383
	STE Between	0.0332	0.2077	0.0267	0.0286
	p-value	4.5e-19	4.1e-6	7.8e-26	0.4268
**Scale 500**					
	Mean Within	0.9090	0.4990	0.8982	0.0395
	Mean Between	0.7261	0.2367	0.6909	0.0229
	STE Within	0.0280	0.1373	0.0263	0.0327
	STE Between	0.0338	0.1290	0.0224	0.0241
	p-value	1.0e-21	1.4e-6	4.6e-33	0.0864

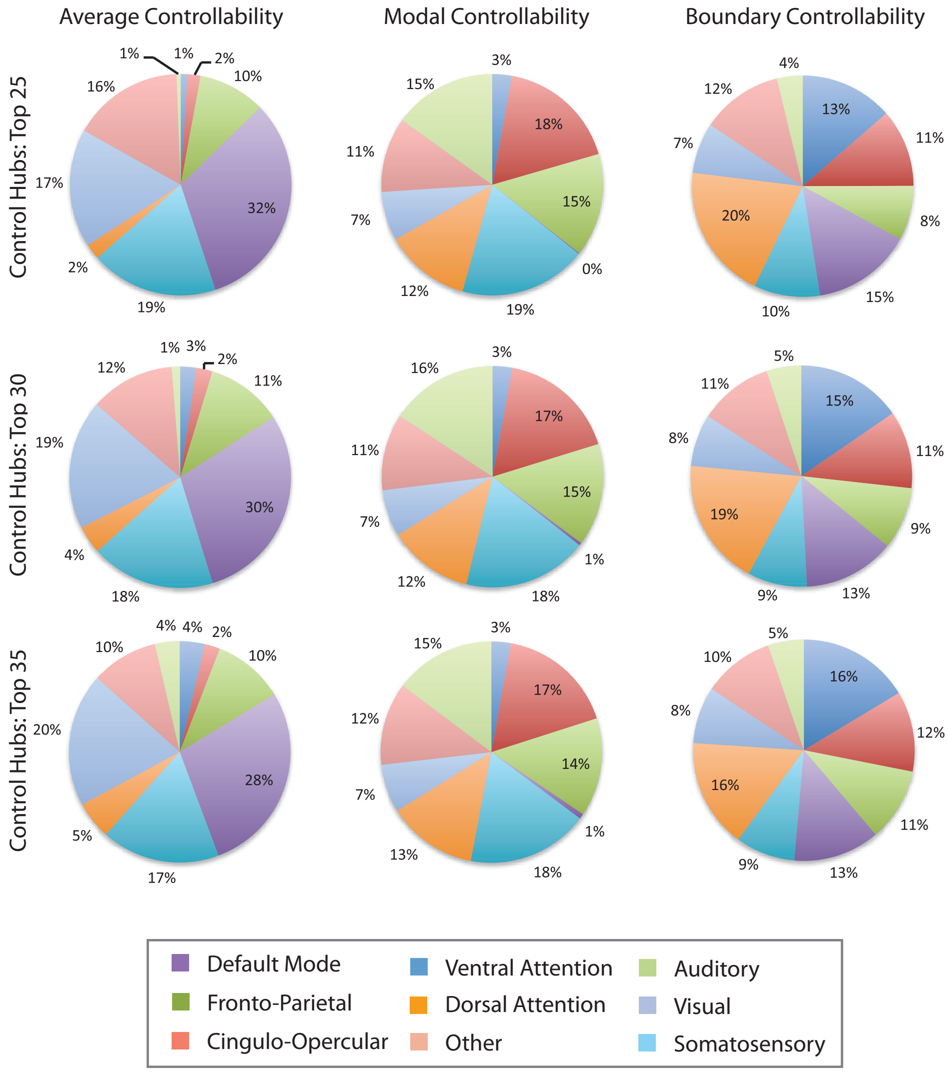

Supplementary Figure 6: **Control Roles of Cognitive Systems.** Cognitive control hubs are differentially located across cognitive systems. *(Left)* Hubs of average controllability are preferentially located in the default mode system. *(Middle)* Hubs of modal controllability are predominantly located in cognitive control systems, including both the fronto-parietal and cingulo-opercular systems. *(Right)* Hubs of boundary controllability are distributed throughout all systems, with the two predominant systems being ventral and dorsal attention systems. These anatomical distributions are consistent across different definitions of control hubs as either *(Top)* the 25 nodes with the highest control values, *(Middle)* the top 30 nodes with the highest control values, or *(Bottom)* the 35 nodes with the highest control values.

by different cognitive systems. In particular, we observed that regions of the default mode system form strong average controllability hubs but weaker modal and boundary controllability hubs. Regions of the cognitive control networks (fronto-parietal and cingulo-opercular) form strong modal controllability hubs and regions of the attentional control networks (ventral and dorsal) form strong boundary controllability hubs. Here we demonstrate that these results are qualitatively reproduced for different definitions of control hubs: namely, the 25 nodes with the highest control values, the 30 nodes with the highest control values (as shown in the main manuscript), or the 35 nodes with the highest control values; see Fig. 7.

In the main text, we validate this finding by performing a repeated measures 2-way Analysis of Variance (ANOVA) with system and controllability diagnostic as categorical factors, and with scan replicate as a repeated measure. Here, we performed the same ANOVA for the case in which control nodes are defined as the 25 nodes with the highest control values or the 35 nodes with the highest control values, and found similar results in both cases: (i) for top 25 nodes, the main effect of system is $F(9) = 43.7716$ ($p = 0$), the main effect of diagnostic is $F(2) = 16.5413$ ($p = 2.0553e-4$), and the interaction between system and diagnostic is $F(18) = 42.1475$ ($p = 0$); (ii) for the top 35 nodes, the main effect of system is $F(9) = 34.3787$ ($p = 0$), the main effect of diagnostic is $F(2) = 9.7420$ ($p = 0.0022$), and the interaction between system and diagnostic is $F(18) = 36.9762$ ($p = 0$). Consistent with the results reported in the main manuscript, these statistics indeed suggest that structural differences between the default mode, cognitive control, and attentional control systems may facilitate their distinct roles in controlling dynamic trajectories of brain network function. We observe that as the number of weaker control hubs we include in the analysis is increased (i.e., larger number of control hubs), the less significant the relationship to cognitive systems. This suggests that the strong control hubs are significantly associated with cognitive systems but that weak control nodes may not be.

Robustness of Results to Alternative Weighting Schemes There is currently no accepted weighting scheme for constructing anatomical networks from diffusion imaging data. Weighting connections between ROIs based on the number of streamlines connecting them (as estimated by diffusion tractography algorithms) is the most commonly utilized scheme. However, it has been argued that these estimates can be biased by variation in the sizes of the regions under study [49]: large regions may have a higher probability of displaying more streamlines than smaller regions. While this potential bias does not appear to drastically alter large-scale topological properties of anatomical networks, its local effects are not well characterized [105]. Our results, based on the number of streamlines, are unlikely to be affected by this potential bias for one key reason: the Lausanne atlas family purposefully attempts to equalize region size, particularly in the higher scales [106]. Nevertheless, to confirm that our results were robust to an alternative weighting scheme that accounts for region size, we divided each ij^{th} element in the adjacency matrix $\mathbf{A}$ in Scale 125 (N=234) by the sum of the sizes of the two regions that it connects to create an alternative adjacency matrix $\mathbf{A}'$. We then computed the controllability diagnostics and rank degree of $\mathbf{A}'$ for each scan. Similar to our results obtained with the original weighting scheme, we observed that (i) the mean average con-

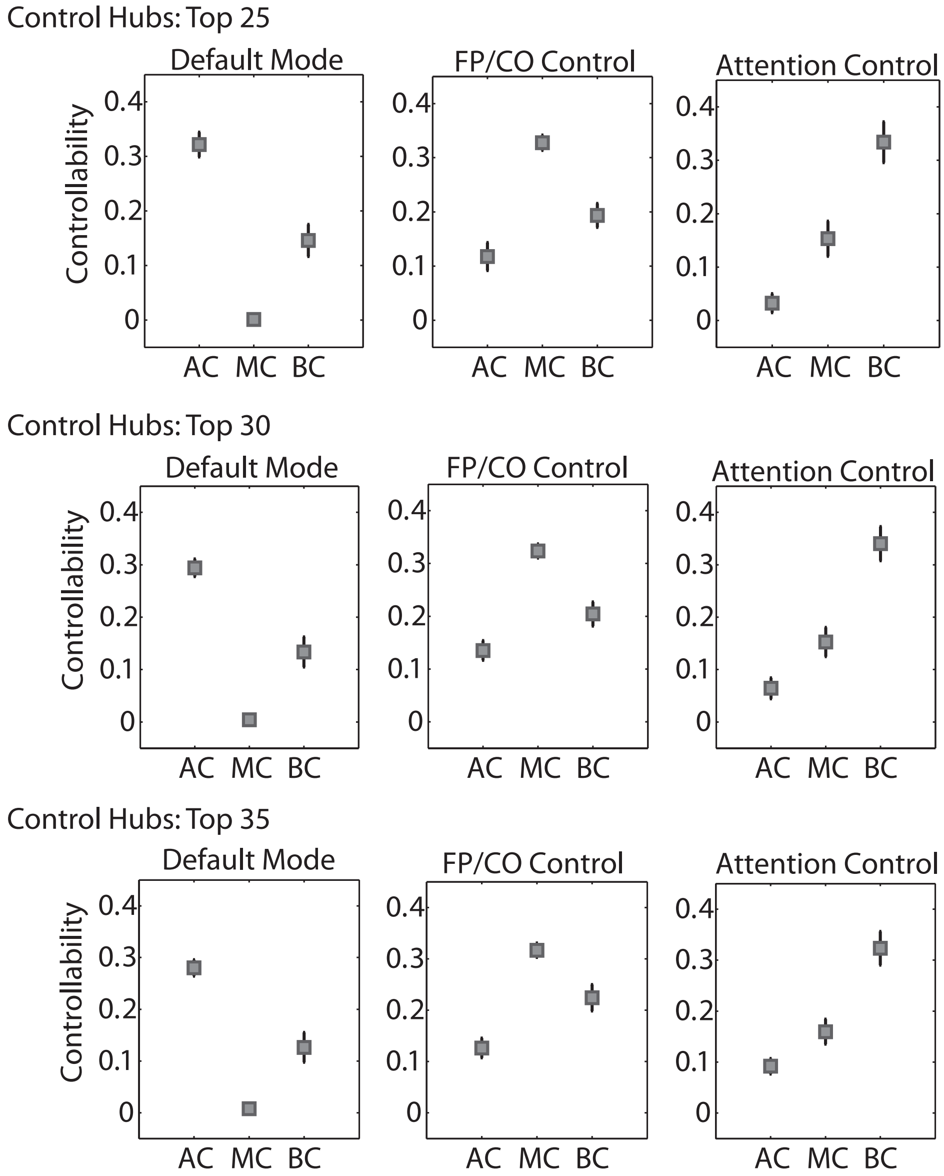

Supplementary Figure 7: **Differential Recruitment of Cognitive Systems to Network Control.** Average controllability (AC), modal controllability (MC), and boundary controllability (BC) hubs are differentially located in default mode *(A)*, fronto-parietal and cingulo-opercular cognitive control *(B)*, and attentional control *(C)* systems. These results are consistently observed whether we define control hubs as the 25 nodes with the highest control values *(Top Row)*, the 30 nodes with the highest control values *(Middle Row)*, or the 35 nodes with the highest control values *(Bottom Row)*. Values are averaged over the 3 replicates for each individual; error bars indicate standard deviation of the mean over subjects.

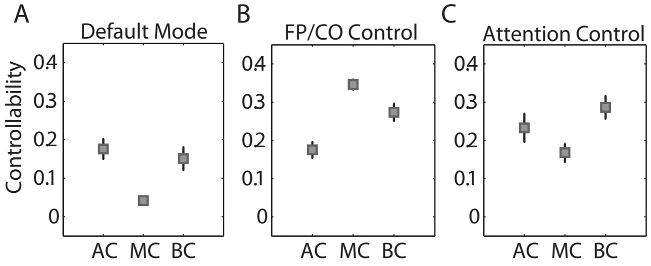

Supplementary Figure 8: **Differential Recruitment of Cognitive Systems to Network Control** Average controllability (AC), modal controllability (MC), and boundary controllability (BC) hubs are differentially located in default mode *(A)*, fronto-parietal and cingulo-opercular cognitive control *(B)*, and attentional control *(C)* systems. Values are averaged over the 3 replicates for each individual; error bars indicate standard deviation of the mean over subjects.

trollability across the scans is strongly and positively correlated with mean rank degree ($r = 0.97$, $p = 1.43 \times 10^{-150}$), (ii) the mean modal controllability across the scans is strongly and negatively correlated with mean rank degree ($r = -0.96$, $p = 2.51 \times 10^{-130}$), and (iii) the mean boundary controllability is not significantly correlated with mean rank degree ($r = -0.01$, $p = 0.32$). Furthermore, the three network controllability diagnostics are again differentially recruited to known cognitive systems in the same manner as they were for the original weighting scheme (Compare Fig. 8 to Fig. 5 in the main manuscript). Together, these findings indicate that the results reported in the main manuscript are robust to variations in weighting scheme that include a correction for region size.

There are two additional considerations that further support the claim that our results cannot be attributed to region size. First, we examine the robustness of our findings across a family of parcellations that vary both in coarseness and in the similarity/disimilarity of region size. The smallest scale Lausanne atlas has some differences in region size, while the finer scale atlases increasingly subdivide larger areas in an effort to create parcellations with similarly sized regions [106]. The robustness of our results in the finer atlases suggests that our results are unlikely to be driven by region size. Second, we directly calculated the Pearson correlation r-squared values between controllability diagnostics and region size (in terms of number of voxels). In scale 125 ($N = 234$ regions) where we report the majority of our results in the main manuscript, we observed that region size accounted for 5.65% of the variability in average controllability, 6.89% of the variability in modal controllability, and 1.17% of the variability in boundary controllability. These results again suggest that region size is not an appreciable driver of controllability diagnostics.

In addition to clarifying that the results reported using the number of streamlines are unlikely to be driven by the size of regions, it is of interest to consider other potential definitions of edge weight. Here we examine two additional edge weight definitions and show that our results are robustly observed in these other weighting schemes. We examine *generalized fractional anisotropy* (GFA) and *quantitative anisotropy* (QA). GFA was defined by Tuch et al. (2004) as an anisotropy

Supplementary Table 4: **Reliability of Controllability Diagnostics Across Edge Weight Definitions** Pearson correlation coefficients between controllability diagnostics extracted for structural brain networks whose edges are weighted by the number of streamlines (SL) and controllability diagnostics extracted for structural brain networks whose edges are weighted by either quantitative anisotropy (QA) or generalized fractional anisotropy (GFA). Controllability diagnostics are abbreviated as follows: average controllability (AC), boundary controllability (BC), modal controllability (MC). Note that for the small system sizes (Scale 33 and Scale 60), all regions are identified as having the same boundary controllability for the choice of parameters $\gamma = 1.6$ and $\rho = 2$; therefore, correlations between these variables in the different edge weighting schemes are not estimable (N/A).

	AC	MC	BC
**Scale 33**			
SL vs. QA	$r = 0.67, p = 1.2 \times 10^{-11}$	$r = 0.66, p = 1.23 \times 10^{-11}$	NA
SL vs. GFA	$r = 0.67, p = 5.3 \times 10^{-12}$	$r = 0.68, p = 1.93 \times 10^{-12}$	NA
**Scale 60**			
SL vs. QA	$r = 0.64, p = 4.0 \times 10^{-16}$	$r = 0.57, p = 1.7 \times 10^{-12}$	NA
SL vs. GFA	$r = 0.64, p = 4.1 \times 10^{-16}$	$r = 0.59, p = 3.0 \times 10^{-13}$	NA
**Scale 125**			
SL vs. QA	$r = 0.64, p = 6.1 \times 10^{-28}$	$r = 0.56, p = 4.3 \times 10^{-21}$	$r = 0.25, p = 1.1 \times 10^{-4}$
SL vs. GFA	$r = 0.62, p = 1.2 \times 10^{-26}$	$r = 0.58, p = 4.5 \times 10^{-22}$	$r = 0.25, p = 1.4 \times 10^{-4}$
**Scale 250**			
SL vs. QA	$r = 0.59, p = 5.4 \times 10^{-45}$	$r = 0.49, p = 5.1 \times 10^{-29}$	$r = 0.12, p = 0.0085$
SL vs. GFA	$r = 0.58, p = 9.0 \times 10^{-43}$	$r = 0.50, p = 1.4 \times 10^{-30}$	$r = 0.17, p = 1.78 \times 10^{-4}$
**Scale 500**			
SL vs. QA	$r = 0.58, p = 7.3 \times 10^{-92}$	$r = 0.58, p = 5.9 \times 10^{-92}$	$r = 0.19, p = 1.6 \times 10^{-9}$
SL vs. GFA	$r = 0.57, p = 5.0 \times 10^{-88}$	$r = 0.59, p = 7.6 \times 10^{-96}$	$r = 0.13, p = 2.3 \times 10^{-5}$

measure on an ODF Ψ:

$$GFA = \frac{\text{std}(\Psi)}{\text{rms}(\Psi)} = \sqrt{\frac{n \sum_{i=1}^{n} \left(\Psi(\mathbf{u}_i) - \langle\Psi\rangle\right)^2}{(n-1)\sum_{i=1}^{n} \Psi(\mathbf{u}_i)^2}} \tag{14}$$

where $\langle\Psi\rangle$ is the mean of the ODF [107]. This measurement produces a single value between [0,1] for each voxel and does not reflect any specific ODF peak. For each streamline, we extracted the average gfa value from all of the voxels the streamline passed through. We then averaged this value over all streamlines that connected a pair of regions, and used this quantity to weight the edge connecting those regions. On the resultant $N \times N$ GFA matrix, we computed the average, modal, and boundary controllability. We observed that the controllability values extracted from the GFA matrix are significantly correlated with the controllability values extracted from the matrices weighted by the number of streamlines (see Table 4). These results indicate that weighting edges by number of streamlines and weighting edges by GFA provide consistent estimates of regional controllability diagnostic values.

Next, we examined the effects of weighting edges by the quantitavive anisotropy. QA is described by Yeh et. al (2010) as a measurement of the signal strength for a specific fiber population $\hat{a}$ in an ODF $\Psi(\hat{a})$ [108]. QA is given by the difference between $\Psi(\hat{a})$ and the isotropic component of the spin density function (SDF, ψ) ISO (ψ) scaled by the SDF's scaling constant. QA differs from GFA in two important ways. First, QA is extracted from the SDF, which is not normalized like the ODF to sum to 1. Therefore QA will be impacted by scanning choices such as echo time. Second, along-streamline QA was calculated based on the angles actually used when tracking each streamline, whereas along-streamline GFA averages values based on the entire ODF which may or may not reflect the signal strength corresponding to the peak used during tracking. Although along-streamline QA is more specific to the anatomical structure being tracked, QA is more sensitive to MRI artifacts such as B1 inhomogeneity.

QA is calculated for each streamline. We then averaged values over all streamlines connecting a pair of regions, and used this value to weight the edge between the regions. On the resultant $N \times N$ QA matrix, we computed the average, modal, and boundary controllability. We observed that the controllability values extracted from the QA matrix are significantly correlated with the controllability values extracted from the matrices weighted by the number of streamlines (see Table 4). These results indicate that weighting edges by number of streamlines and weighting edges by QA provide consistent estimates of regional controllability diagnostic values.

Robustness of Results to Alternative Imaging Acquisitions and Independent Subject Cohorts
In the main manuscript, we show that average control hubs are differentially associated with the default mode system, modal control hubs are differentially associated with fronto-parietal and cingulo-opercular systems, and boundary control hubs are differentially associated with dorsal and ventral attention systems. Here we show that these observations are robust to the choice of imaging acquisition and to independent subject cohorts. Using a diffusion tensor imaging data set of 85 healthy adult human subjects (see Supplementary Methods), we again find that regions of high controllability are differentially associated with the 8 cognitive systems (see Fig. 9). Consistent with the analysis outlined in the main manuscript, we define the set of high control hubs as the 30 regions with the largest controllability values (averaged over all scans), and we calculate the percent of hubs present from each of the 8 cognitive systems. To correct for system size, we normalize the raw percentage of hubs present in a given cognitive system by the number of regions in a cognitive system. By applying this normalization, systems composed of a larger number of regions do not have a greater or lesser chance of housing one of the top 30 control hubs than systems composed of a smaller number of regions. Consistent with the results obtained from DSI data, 32% of average control hubs lie in the default mode system (compare to 30% reported in the main manuscript), 31% of modal control hubs lie in the fronto-parietal and cingulo-opercular cognitive control systems (compare to 32% reported in the main manuscript), and 33% of boundary control hubs lie in the ventral and dorsal attention systems (compare to 34% reported in the main manuscript). These results demonstrate that the differential association of control hubs to these systems is consistent in both diffusion spectrum imaging and diffusion tensor imaging, and is

robustly observed across independent samples of drastically different sizes ($N = 8$ for the DSI data and $N = 85$ for the DTI data).

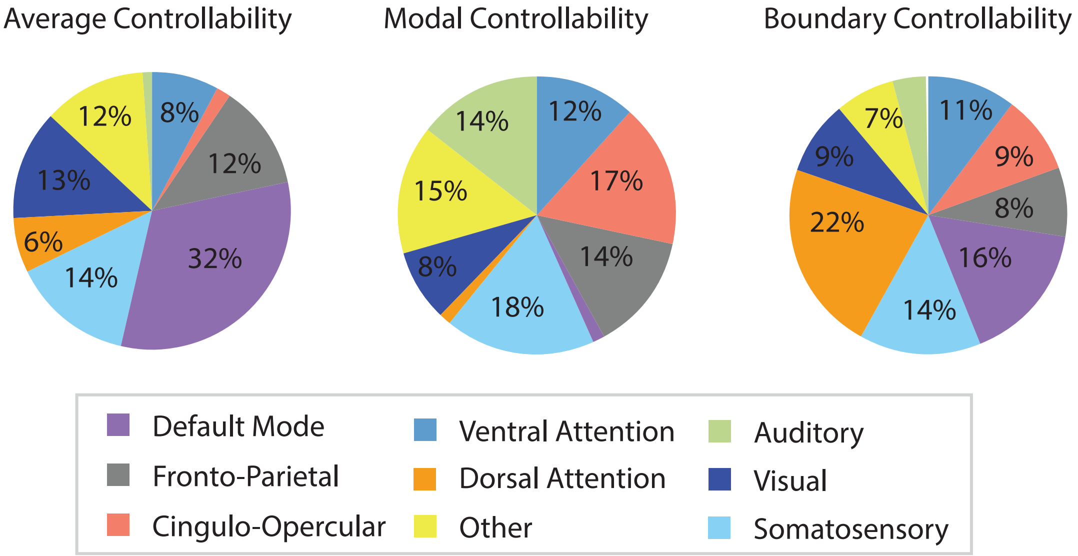

Supplementary Figure 9: **Control Roles of Cognitive Systems Extracted from DTI Data** Cognitive control hubs are differentially located across cognitive systems. *(A)* Hubs of average controllability are preferentially located in the default mode system. *(B)* Hubs of modal controllability are predominantly located in cognitive control systems, including both the fronto-parietal and cingulo-opercular systems. *(C)* Hubs of boundary controllability are distributed throughout all systems, with the two predominant systems being ventral and dorsal attention systems. Control hubs have been identified at the group level as the 30 regions with the highest controllability values (averaged over subjects). Raw percentages of control hubs present in each system have been normalized by the number of regions in the cognitive system. By applying this normalization, systems composed of a larger number of regions have the same chance of housing one of the top 30 control hubs as systems composed of a smaller number of regions.

In the main manuscript, we further show that there is a significant controllability-by-system interaction using structural brain networks extracted from DSI data in 8 healthy controls. Here we show that these results are robustly observed in a different imaging scheme and in a large independent subject cohort. Using a diffusion tensor imaging data set of 85 healthy adult human subjects (see Supplementary Methods), we again extract control hubs for each scan, determine their association with the three hypothesized control systems (default mode, fronto-parietal and cingulo-opercular cognitive control, and attentional control), and quantify the mean controllability value for all hubs in each system (Fig 10). We again observe that regions of the default mode system form strong average controllability hubs but weaker modal and boundary controllability hubs. Regions of the cognitive control networks (fronto-parietal and cingulo-opercular) form strong modal controllability hubs and regions of the attentional control networks (ventral and dorsal) form strong boundary controllability hubs. These results confirm in a different imaging acquisition and large independent cohort of subjects that structural differences between the default mode, cognitive control, and attentional control systems may facilitate their distinct roles in controlling trajectories of

brain network function.

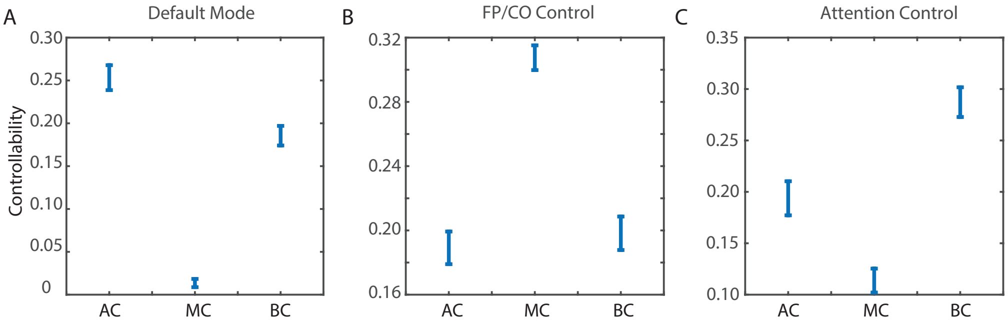

Supplementary Figure 10: **Differential Recruitment of Cognitive Systems to Network Control Extracted from DTI Data** Average controllability (AC), modal controllability (MC), and boundary controllability (BC) hubs are differentially located in default mode *(A)*, fronto-parietal and cingulo-opercular cognitive control *(B)*, and attentional control *(C)* systems. Error bars indicate standard deviation of the mean over subjects.

Controllability in Cortical vs. Whole-Brain Circuitry In the main manuscript, we describe results for a whole-brain network that includes cortical and subcortical structures. It is of interest to also examine the controllability of cortical circuitry alone, as this is a common object of interest in some areas of systems neuroscience. Moreover, it remains an open question whether subcortical structures are best included in whole-brain parcellations as single entities (as utilized in this work and other recent studies), or subdivided into relevant nuclei (which trades the benefits of anatomical specificity for the disadvantages of signal-to-noise ratio). To examine controllability in cortical circuitry, we therefore removed subcortical regions of interest from our analysis (thalamus, caudate, putamen, pallidum, nucleus accumbens, hippocampus, and amygdala), and computed a new adjacency matrix $\mathbf{A}_{\text{cortical}}$. Using this adjacency matrix, we recalculated the values of controllability diagnostics for the Scale 125 Lausanne atlas, and show results of this analysis in Fig. 11. We observe that the results reported in the main manuscript remain qualitatively conserved: average controllability is positively correlated with weighted degree, modal controllability is negatively correlated with weighted degree, and boundary controllability is less well correlated with weighted degree.

We also identify the control roles of cognitive systems estimated by this cortical circuitry (see Fig. 12). Here we observe, similarly to the results reported in the main manuscript for the whole-brain circuitry, that control hubs are distributed throughout cortical systems, but are over-represented in a few specific systems. Modal control hubs, defined as the 30 regions with the highest modal controllability, are over-represented in cognitive control systems – fronto-parietal and cingulo-opercular – which account for 32% of the control hubs, identical to the 32% observed in the whole-brain circuitry. Boundary control hubs are over-represented in the attentional control

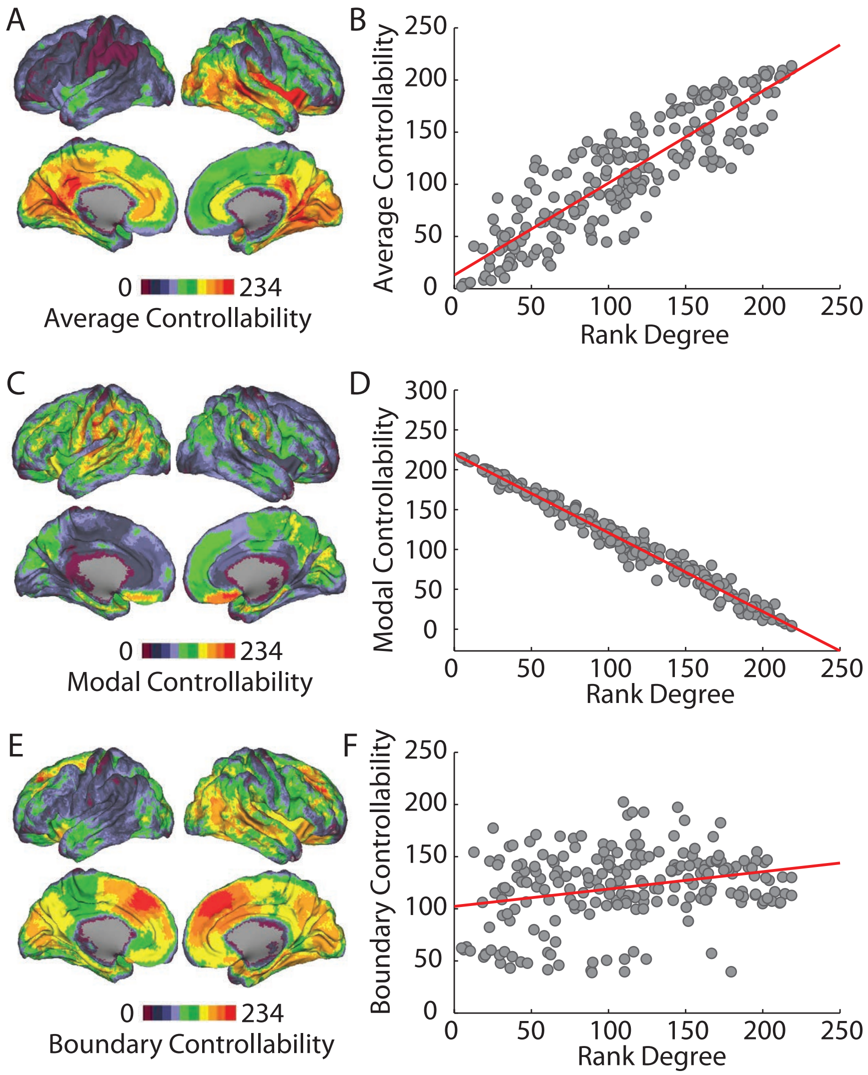

Supplementary Figure 11: **Brain Network Control Properties for Cortical Circuitry** *(A)* Average controllability quantifies control to many states. Here we show average controllability values ranked for all brain regions plotted on a surface visualization. Warmer colors indicate larger values of average controllability. *(B)* Scatter plot of weighted degree (ranked for all brain regions) versus average controllability (Pearson correlation $r = 0.88$, $p = 2 \times 10^{-73}$). *(C)* Modal controllability quantifies control to difficult-to-reach states. Here we show modal controllability values ranked for all brain regions plotted on a surface visualization. *(D)* Scatter plot of weighted degree (ranked for all brain regions) versus modal controllability ($r = -0.99$, $p = 1 \times 10^{-180}$). *(E)* Boundary controllability quantifies control to decouple or integrate network modules. Here we show boundary controllability values ranked for all brain regions plotted on a surface visualization. *(F)* Scatter plot of weighted degree (ranked for all brain regions) versus boundary controllability ($r = 0.27$, $p = 6 \times 10^{-5}$). In panels *(A)*, *(C)*, and *(E)*, warmer colors indicate larger controllability values, which have been averaged over both replicates and subjects.

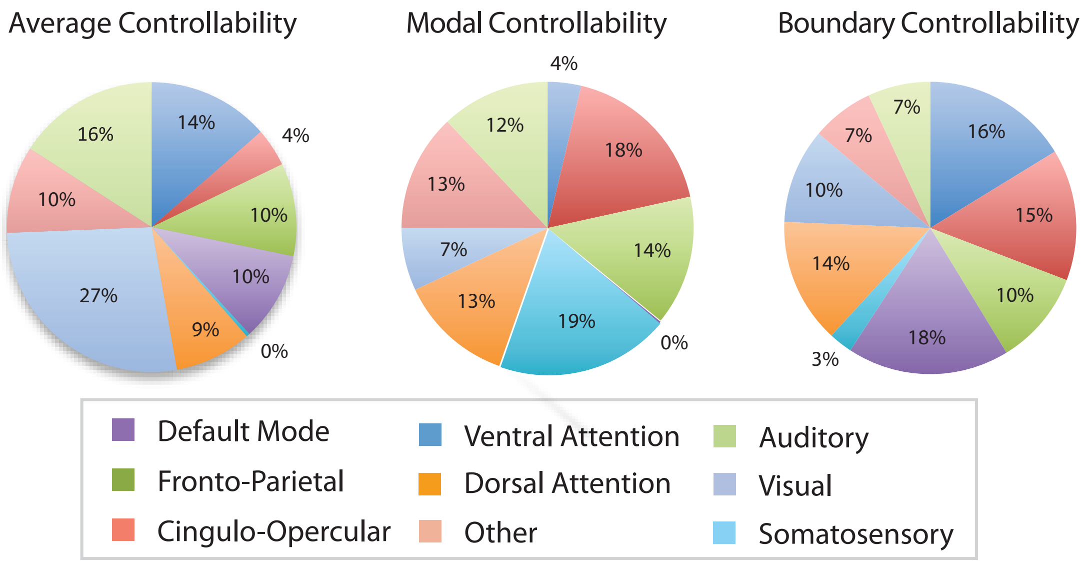

Supplementary Figure 12: **Control Roles of Cognitive Systems for Cortical Circuitry** Cognitive control hubs are differentially located across cognitive systems. *(A)* Hubs of average controllability are preferentially located in the default mode system. *(B)* Hubs of modal controllability are predominantly located in cognitive control systems, including both the fronto-parietal and cingulo-opercular systems. *(C)* Hubs of boundary controllability are distributed throughout all systems, with the two predominant systems being ventral and dorsal attention systems. Control hubs have been identified at the group level as the 30 regions with the highest controllability values (averaged over replicates and subjects).

systems – dorsal and ventral attention systems – which account for 30% of control hubs compared with 34% in the whole-brain circuitry. These results confirm the findings reported in the main manuscript for the whole-brain circuitry. The top 30 average control hubs, however, show a different distribution across cognitive systems in the cortical circuitry as opposed to the whole-brain circuitry. Specifically, in the whole-brain circuitry, we observed an over-representation of the default mode system in average control hubs (30% of top 30) which is not conserved in the cortical circuitry alone (10% of top 30). However, if we examine the strongest average control hubs (5 regions with the highest average controllability values), we observe that 25% of them lie in the default mode system.

Finally, we also examined the differential recruitment of cognitive systems to network control in the cortical versus whole-brain circuitry. In Fig. 13, we show the controllability values of control hubs in the default mode, cognitive control, and attentional control systems. Consistent with the results reported in the main manuscript for the whole-brain circuitry, the cortical circuitry shows that regions of the cognitive control systems form strong modal controllability hubs, while regions of the attentional control systems form strong boundary controllability hubs. In contrast to the results reported in the main manuscript for whole-brain circuitry, the cortical circuitry alone shows that regions of the default mode system are not the strongest average controllability hubs when

hubs are defined as the 30 regions with the largest values of controllability. However, if we confine ourselves to examining only the 5 strongest average controllability hubs, we recover the finding that regions of the default mode system form strong average controllability hubs (mean controllability value 0.24, STD 0.10). These results indicate that the strongest average controllability hubs remain in the default mode system, consistent with the results reported in the main manuscript.

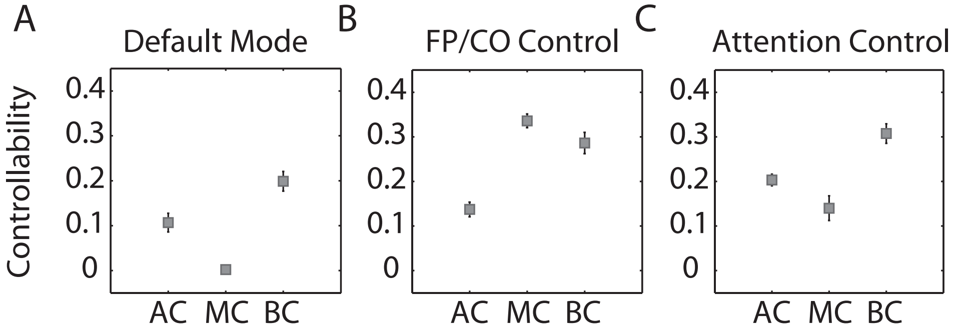

Supplementary Figure 13: **Differential Recruitment of Cognitive Systems to Network Control for Cortical Circuitry** Average controllability (AC), modal controllability (MC), and boundary controllability (BC) hubs are differentially located in default mode *(A)*, fronto-parietal and cingulo-opercular cognitive control *(B)*, and attentional control *(C)* systems. Values are averaged over the 3 replicates for each individual; error bars indicate standard deviation of the mean over subjects.

Relationships Between Controllability Diagnostics and Other Network Variables In the main manuscript, we examine the relationship between controllability diagnostics and the weighted degree (also often referred to as *strength*). Degree is more generally thought of as a measure of centrality, influence, or importance and it may be of interest to examine how controllability diagnostics relate to other measures of centrality. Here, we compare controllability diagnostic values to a second common measure of centrality: the *betweenness centrality* [109]. The betweenness centrality for a node v in a network was originally defined for binary networks, and measures the proportion of shortest paths between any two nodes i and j that must pass through node v. Here we use a fast algorithm [110] to calculate the *weighted* betweenness centrality for consistency with the weighted analyses utilized in the remainder of our work. As shown in the left-most panels of Fig. 14, the average and boundary controllability are positively correlated with the betweenness centrality (Pearson correlation coefficient $r = 0.42$, $p = 1 \times 10^{-11}$ and $r = 0.28$, $p = 1 \times 10^{-5}$, respectively), while the modal controllability is negatively correlated with the betweenness centrality ($r = -0.33$, $p = 1 \times 10^{-7}$). The direction of these relationships is consistent with those reported in the main manuscript for degree, although the strength of the relationships is diminished for modal and average controllability, and increased for boundary controllability, in comparison. These results indicate that different centrality measures (which take into account only structural information in the network) may be differentially correlated with controllability diagnostics (which take into account both structural and dynamic information in the network).

It is also interesting to compare boundary controllability and the participation coefficient [111]. The boundary controllability identifies control nodes present at the boundaries between communities, and does so in a hierarchically descending manner from the largest scale partition of the network into a few communities, to the smallest scale partition of the network into N communities. The participation coefficient measures – for a single large scale partition – the participation of a node in different communities, and takes a value of 1 when the node's links are uniformly distributed among all the communities and a value of 0 when the node's links are all within its own community [111]. In Fig. 14F, we show that the boundary controllability is significantly positively correlated with the participation coefficient (Pearson correlation coefficient $r = 0.39$, $p = 6 \times 10^{-10}$). However, there is significant scatter in this plot, which is consistent with the fact that the participation coefficient examines the community structure at one scale of network organization (a single large scale partition of the network into a few communities), while the boundary controllability examines the community structure across the full hierarchy of scales of network organization. For completeness, relationships between average and modal controllability and participation coefficient are also shown in Fig. 14B and D.

In the context of these results that highlight the relationships between static graph theoretical diagnostics and network controllability diagnostics, it is important to affirm that the two methods differ from one another significantly. While graph statistics utilize only structural connectivity information, network controllability diagnostics utilize both (i) a structural connectivity matrix and (ii) an equation of state defining the dynamics that occur on top of that structure. Therefore, observed relationships between the diagnostics extracted from the two approaches (graph theory and network controllability) cannot be postulated to hold for any dynamical system in any context. Our results must therefore be interpreted solely in the context of the specific structural connectivity and dynamics that we utilize in Equation 1 in the main manuscript.

Common Graphical Metrics of Brain Networks While the focus of this paper lies in examining controllability diagnostics of structural brain networks and their theoretical implications for cognition, it is also of interest to describe the basic architectural properties of these networks. In Table 6, we provide mean and standard error of common graph diagnostics, including degree, path length, clustering coefficient, modularity index, global efficiency, and density calculated for the DSI-based structural networks weighted by the number of white matter streamlines across the Lausanne atlas parcellation family. For comparison, we also provide a similar table (Table 4) containing the mean and standard error of the same graph diagnostics calculated for random network controls that maintain the same strength distribution as the real networks. We observe that these networks (i) are on average quite sparse, with weighted densities ranging from 0.6 (Scale 33) to 0.1 (Scale 500), and (ii) display a longer path length, greater clustering, decreased global efficiency, and greater modularity than their random network counterparts. These results are consistent with previous studies in similar structural brain networks [49,105].

Supplementary Table 5: **Means and Standard Errors of Common Graph Diagnostics Estimated on Structural Brain Networks** Graph diagnostic values are calculated on structural brain networks estimated from DSI data, and weighted by number of streamlines. Values are averaged over brain regions, subjects, and scans; standard errors are normalized by $\sqrt{number\_of\_subjects}$. Diagnostics include degree (DG), path length (PL), clustering coefficient (CC), modularity index (MD), global efficiency (GE), and density (DS). Calculations are performed using the Brain Connectivity Toolbox [112].

	DG	PL	CC	MD	GE	DS
Scale 33	49.27 ± 1.65	3.07 ± 0.03	195.12 ± 4.04	0.33 ± 0.01	735.55 ± 10.69	0.60 ± 0.02
Scale 60	61.03 ± 2.23	3.51 ± 0.03	122.87 ± 3.92	0.38 ± 0.01	508.49 ± 6.68	0.48 ± 0.02
Scale 125	74.92 ± 3.06	4.00 ± 0.02	73.71 ± 2.41	$0.43pm0.01$	325.92 ± 4.77	0.32 ± 0.01
Scale 250	87.08 ± 4.17	4.44 ± 0.02	43.48 ± 1.67	0.48 ± 0.01	191.41 ± 3.93	0.19 ± 0.01
Scale 500	97.80 ± 5.59	4.77 ± 0.03	25.30 ± 0.88	0.55 ± 0.01	101.96 ± 3.01	0.10 ± 0.01

Supplementary Table 6: **Means and Standard Errors of Common Graph Diagnostics Estimated on Random Null Model Networks** The connections in true brain networks are redistributed throughout the network while maintaining the strength distributions using the code $\mathrm{randmio_und.m}$ in the Brain Connectivity Toolbox [112]. Graph diagnostic values are then calculated and averaged over brain regions, subjects, and scans; standard errors are normalized by $\sqrt{number\_of\_subjects}$. Diagnostics include degree (DG), path length (PL), clustering coefficient (CC), modularity (MD), global efficiency (GE), and density (DS). Calculations are performed using the Brain Connectivity Toolbox [112].

	DG	PL	CC	MD	GE	DS
Scale 33	49.27 ± 1.65	2.58 ± 0.02	133.52 ± 1.99	0.21 ± 0.01	947.88 ± 10.14	0.60 ± 0.02
Scale 60	61.03 ± 2.23	2.83 ± 0.02	72.86 ± 1.01	0.21 ± 0.01	648.97 ± 7.93	0.48 ± 0.02
Scale 125	74.92 ± 3.06	3.19 ± 0.02	34.48 ± 0.27	0.21 ± 0.01	$423.17.92 \pm 5.34$	0.32 ± 0.01
Scale 250	87.08 ± 4.17	3.56 ± 0.02	15.50 ± 0.19	0.21 ± 0.01	262.23 ± 3.23	0.19 ± 0.01
Scale 500	97.80 ± 5.58	3.86 ± 0.03	6.58 ± 0.11	0.20 ± 0.01	155.60 ± 2.44	0.10 ± 0.01

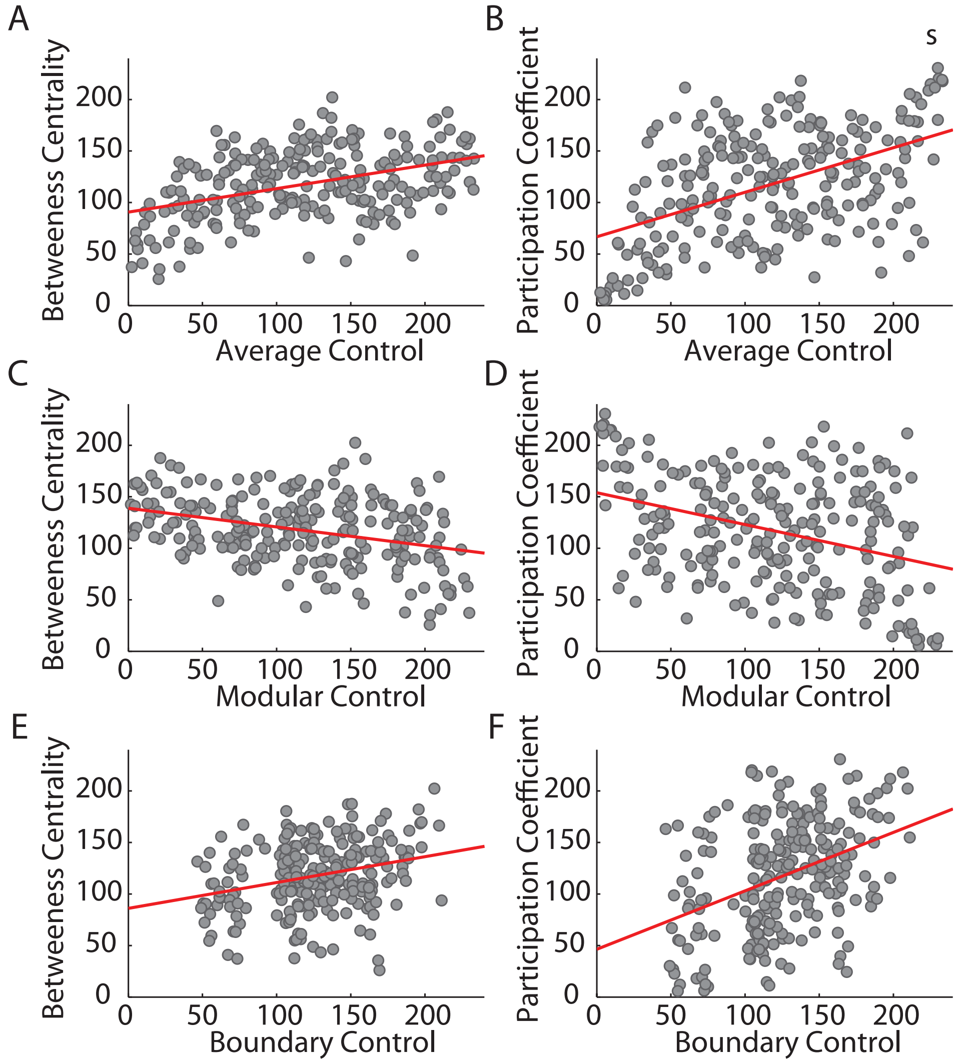

Supplementary Figure 14: **Controllability Diagnostics *versus* Betweenness Centrality and Participation Coefficient** Scatterplots of regional values of average controllability (top panels *(A,B)*), modal controllability (middle panels *(C,D)*), and boundary controllability (bottom panels *(E,F)*) versus regional values of betweenness centrality (left panels *(A,C,E)*) and participation coefficient (right panels *(B,D,F)*). Values are averaged over the 3 replicates for each individual.

Supplementary References

1. Wedeen, V. J., Hagmann, P., Tseng, W. Y. I., Reese, T. G. & Weisskoff, R. M. Mapping complex tissue architecture with diffusion spectrum magnetic resonance imaging. *Magnetic Resonance in Medicine* **54**, 1377–1386 (2005).

2. Yeh, F. C. & Tseng, W. Ntu-90: a high angular resolution brain atlas constructed by q-space diffeomorphic reconstruction. *Neuroimage* **58**, 91–99 (2011).

3. Cieslak, M. & Grafton, S. T. Local termination pattern analysis: a tool for comparing white matter morphology. *Brain Imaging Behav* **8**, 292–299 (2014).

4. Honey, C. J. *et al.* Predicting human resting-state functional connectivity from structural connectivity. *Proc Natl Acad Sci U S A* **106**, 2035–2040 (2009).

5. Fernández Galán, R. On how network architecture determines the dominant patterns of spontaneous neural activity. *PLoS One* **3**, e2148 (2008).

6. Rajapakse, I., Groudine, M. & Mesbahi, M. Dynamics and control of state-dependent networks for probing genomic organization. *Proc. Nat. Acad. Sci. USA* **108**, 17257–17262 (2011).

7. Yan, G., Ren, J., Lai, Y.-C., Lai, C.-H. & Li, B. Controlling complex networks: How much energy is needed? *Physical Review Letters* **108**, 218703 (2012).

8. Sun, J. & Motter, A. E. Controllability transition and nonlocality in network control. *Physical Review Letters* **110**, 208701 (2013).

9. Müller, F.-J. & Sachuppert, A. Few inputs can reprogram biological networks. *Nature* **478**, E4–E4 (2011).

10. Rahmani, A., Ji, M., Mesbahi, M. & Egerstedt, M. Controllability of multi-agent systems from a graph-theoretic perspective. *SIAM Journal on Control and Optimization* **48**, 162–186 (2009).

11. Notarstefano, G. & Parlangeli, G. Controllability and observability of grid graphs via reduction and symmetries. *IEEE Transactions on Automatic Control* **58**, 1719–1731 (2013).

12. Goni, J. *et al.* Resting-brain functional connectivity predicted by analytic measures of network communication. *Proc Natl Acad Sci U S A* **111**, 833–838 (2014).

13. Li, Y. *et al.* Brain anatomical network and intelligence. *PLoS Comput Biol* **5**, e1000395 (2009).

14. Griffa, A., Baumann, P. S., Thiran, J. P. & Hagmann, P. Structural connectomics in brain diseases. *Neuroimage* **80**, 515–526 (2013).

15. Fornito, A., Zalesky, A., Pantelis, C. & Bullmore, E. T. Schizophrenia, neuroimaging and connectomics. *Neuroimage* **62**, 2296–2314 (2012).

16. Raj, A., Kuceyeski, A. & Weiner, M. A network diffusion model of disease progression in dementia. *Neuron* (2012, volume = 73, number = 6, pages = 1204–1215,).

17. Cao, Q. *et al.* Probabilistic diffusion tractography and graph theory analysis reveal abnormal white matter structural connectivity networks in drug-naive boys with attention deficit/hyperactivity disorder. *J Neurosci* **33**, 10676–10687 (2013).

18. Honey, C. J., Thivierge, J. P. & Sporns. Can structure predict function in the human brain? *Neuroimage* **52**, 766–776 (2010).

19. Abdelnour, F., Voss, H. U. & Raj, A. Network diffusion accurately models the relationship between structural and functional brain connectivity networks. *Neuroimage* **90**, 335–347 (2014).

20. Brown, J. A. *et al.* Brain network local interconnectivity loss in aging APOE-4 allele carriers. *Proc Natl Acad Sci U S A* **108**, 20760–20765 (2011).

21. Short, S. J. *et al.* Associations between white matter microstructure and infants' working memory. *Neuroimage* **64**, 156–166 (2013).

22. Felleman, D. J. & Van Essen, D. C. Distributed hierarchical processing in the primate cerebral cortex. *Cereb Cortex* **1**, 1–47 (1991).

23. Pasqualetti, F. & Zampieri, S. On the controllability of isotropic and anisotropic networks. In *IEEE Conf. on Decision and Control* (Los Angeles, CA, USA, 2014). To appear.

24. Kailath, T. *Linear Systems* (Prentice-Hall, 1980).

25. Reinschke, K. J. *Multivariable Control: A Graph-Theoretic Approach* (Springer, 1988).

26. Kailath, T. *Linear systems*, vol. 1 (Prentice-Hall Englewood Cliffs, NJ, 1980).

27. Marx, B., Koenig, D. & Georges, D. Optimal sensor and actuator location for descriptor systems using generalized Gramians and balanced realizations. In *American Control Conference*, 2729–2734 (Boston, MA, USA, 2004).

28. Shaker, H. R. & Tahavori, M. Optimal sensor and actuator location for unstable systems. *Journal of Vibration and Control* (2012). URL `http://jvc.sagepub.com/cgi/content/abstract/1077546312451302v1`. `http://jvc.sagepub.com/content/early/2012/07/16/1077546312451302.full.pdf+html`.

29. Summers, T. H. & Lygeros, J. Optimal sensor and actuator placement in complex dynamical networks (2013). ArXiv preprint arXiv:1306.2491.

30. Pasqualetti, F., Zampieri, S. & Bullo, F. Controllability metrics, limitations and algorithms for complex networks. *IEEE Transactions on Control of Network Systems* **1**, 40–52 (2014).

31. Newman, M. E. Modularity and community structure in networks. *Proc Natl Acad Sci U S A* **103**, 8577–8582 (2006).

32. Blondel, V. D., Guillaume, J.-L., Lambiotte, R. & Lefebvre, E. Fast unfolding of communities in large networks. *Journal of Statistical Mechanics: Theory and Experiment* **10**, P1000 (2008).

33. Jutla, I. S., Jeub, L. G. S. & Mucha, P. J. A generalized Louvain method for community detection implemented in MATLAB (2011–2012). URL `http://netwiki.amath.unc.edu/GenLouvain`.

34. Meunier, D., Lambiotte, R. & Bullmore, E. T. Modular and hierarchically modular organization of brain networks. *Front Neurosci* **4**, 200 (2010).

35. Bassett, D. S. & Siebenhuhner, F. *In Multiscale Analysis and Nonlinear Dynamics: From Genes to the Brain*, chap. Multiscale network organization in the human brain (Wiley, 2013).

36. Meunier, D., Achard, S., Morcom, A. & Bullmore, E. Age-related changes in modular organization of human brain functional networks. *Neuroimage* **44**, 715–723 (2009).

37. Chen, Z. J., He, Y., Rosa-Neto, P., Germann, J. & Evans, A. C. Revealing modular architecture of human brain structural networks by using cortical thickness from MRI. *Cereb Cortex* **18**, 2374–2381 (2008).

38. Bassett, D. S. *et al.* Dynamic reconfiguration of human brain networks during learning. *Proc Natl Acad Sci U S A* **108**, 7641–7646 (2011).

39. Power, J. D. *et al.* Functional network organization of the human brain. *Neuron* **72**, 665–678 (2011).

40. Newman, M. E. & Girvan, M. Finding and evaluating community structure in networks. *Phys Rev E* **69**, 026113 (2004).

41. Newman, M. E. Fast algorithm for detecting community structure in networks. *Phys Rev E* **69**, 066133 (2004).

42. Porter, M. A., Onnela, J.-P. & Mucha, P. J. Communities in networks. *Notices of the American Mathematical Society* **56**, 1082–1097, 1164–1166 (2009).

43. Fortunato, S. Community detection in graphs. *Phys Rep* **486**, 75–174 (2010).

44. Brandes, U. *et al.* On modularity clustering. *IEEE Trans on Knowl Data Eng* **20**, 172–188 (2008).

45. Good, B. H., de Montjoye, Y. A. & Clauset, A. Performance of modularity maximization in practical contexts. *Phys Rev E* **81**, 046106 (2010).

46. Reichardt, J. & Bornholdt, S. Statistical mechanics of community detection. *Phys Rev E* **74**, 016110 (2006).

47. Onnela, J.-P. *et al.* Taxonomies of networks from community structure. *Phys Rev E* **86**, 036104 (2012).

48. Traud, A. L., Kelsic, E. D., Mucha, P. J. & Porter, M. A. Comparing community structure to characteristics in online collegiate social networks. *SIAM Review* **53**, 526–543 (2011).

49. Hagmann, P. *et al.* Mapping the structural core of human cerebral cortex. *PLoS Biology* **6**, e159 (2008).

50. Zald, D. H. *et al.* Meta-analytic connectivity modeling reveals differential functional connectivity of the medial and lateral orbitofrontal cortex. *Cereb Cortex* **24**, 232–248 (2014).

51. Rothkirch, M., Schmack, K., Schlagenhauf, F. & Sterzer, P. Implicit motivational value and salience are processed in distinct areas of orbitofrontal cortex. *Neuroimage* **62**, 1717–1725 (2012).

52. Anderson, J. S., Ferguson, M. A., Lopez-Larson, M. & Yurgelun-Todd, D. Topographic maps of multisensory attention. *Proc Natl Acad Sci U S A* **107**, 20110–20114 (2010).

53. Xiang, H. D., Fonteijn, H. M., Norris, D. G. & Hagoort, P. Topographical functional connectivity pattern in the perisylvian language networks. *Cereb Cortex* **20**, 549–560 (2010).

54. Egner, T. Right ventrolateral prefrontal cortex mediates individual differences in conflict-driven cognitive control. *J Cogn Neurosci* **23**, 3903–3913 (2011).

55. Enriquez-Geppert, S. *et al.* Functional parcellation of the inferior frontal and midcingulate cortices in a flanker-stop-change paradigm. *Hum Brain Mapp* **34**, 1501–1514 (2013).

56. Elton, A. & Gao, W. Divergent task-dependent functional connectivity of executive control and salience networks. *Cortex* **51**, 56–66 (2014).

57. Tu, P. C., Lee, Y. C., Chen, Y. S., Li, C. T. & Su, T. P. Schizophrenia and the brain's control network: aberrant within- and between-network connectivity of the frontoparietal network in schizophrenia. *Schizophr Res* **147**, 339–347 (2013).

58. Stern, E. R., Fitzgerald, K. D., Welsh, R. C., Abelson, J. L. & Taylor, S. F. Resting-state functional connectivity between fronto-parietal and default mode networks in obsessive-compulsive disorder. *PLoS One* **7**, e36356 (2012).

59. Xu, C. P. *et al.* Altered functional connectivity within and between brain modules in absence epilepsy: a resting-state functional magnetic resonance imaging study. *Biomed Res Int* 734893 (2013).

60. Lukoshe, A., White, T., Schmidt, M. N., van der Lugt, A. & Hokken-Koelega, A. C. Divergent structural brain abnormalities between different genetic subtypes of children with Prader-Willi syndrome. *J Neurodev Disord* **5**, 31 (2013).

61. Sun, H. *et al.* Abnormal activity of default mode network in GERD patients. *BMC Neurosci* **14**, 69 (2013).

62. Fang, T. *et al.* Functional connectivity changes in patients with absence epilepsy studied using resting-state functional MRI. *J Clin Neurosci* **20**, 413–418 (2013).

63. Marqués-Iturria, I. *et al.* The interaction effect between BDNF val66met polymorphism and obesity on executive functions and frontal structure. *Am J Med Genet B Neuropsychiatr Genet* **165**, 245–253 (2014).

64. Lopez-Larson, M. P. *et al.* Altered prefrontal and insular cortical thickness in adolescent marijuana users. *Behav Brain Res* **220**, 164–172 (2011).

65. Durazzo, T. C. *et al.* Cortical thickness, surface area, and volume of the brain reward system in alcohol dependence: relationships to relapse and extended abstinence. *Alcohol Clin Exp Res* **35**, 1187–1200 (2011).

66. Verstraete, E., Veldink, J. H., Mandl, R. C., van den Berg, L. H. & van den Heuvel, M. P. Impaired structural motor connectome in amyotrophic lateral sclerosis. *PLoS One* **6**, e24239 (2011).

67. Duffield, T. C. *et al.* Neuropsychological investigation of motor impairments in autism. *J Clin Exp Neuropsychol* **35**, 867–881 (2013).

68. Ninaus, M. *et al.* Neural substrates of cognitive control under the belief of getting neurofeedback training. *Front Hum Neurosci* **7**, 914 (2013).

69. Gratton, C., Lee, T. G., Nomura, E. M. & D'Esposito, M. The effect of theta-burst TMS on cognitive control networks measured with resting state fMRI. *Front Syst Neurosci* **7**, 124 (2013).

70. Vaden, K. I. J. *et al.* The cingulo-opercular network provides word-recognition benefit. *J Neurosci* **33**, 18979–18986 (2013).

71. Becerril, K. E. & Barch, D. M. Conflict and error processing in an extended cingulo-opercular and cerebellar network in schizophrenia. *Neuroimage Clin* **3**, 470–480 (2013).

72. Sestieri, C., Corbetta, M., Spadone, S., Romani, G. L. & Shulman, G. L. Domain-general signals in the cingulo-opercular network for visuospatial attention and episodic memory. *J Cogn Neurosci* **26**, 551–568 (2014).

73. Tu, P. C., Hsieh, J. C., Li, C. T., Bai, Y. M. & Su, T. P. Cortico-striatal disconnection within the cingulo-opercular network in schizophrenia revealed by intrinsic functional connectivity analysis: a resting fMRI study. *Neuroimage* **59**, 238–247 (2012).

74. Khalsa, S., Mayhew, S. D., Chechlacz, M., Bagary, M. & Bagshaw, A. P. The structural and functional connectivity of the posterior cingulate cortex: Comparison between deterministic and probabilistic tractography for the investigation of structure-function relationships. *Neuroimage* **S1053–8119**, 01226–01233 (2013).

75. Leech, R. & Sharp, D. J. The role of the posterior cingulate cortex in cognition and disease. *Brain* **137(Pt 1)**, 12–32 (2014).

76. Zhu, D. C., Majumdar, S., Korolev, I. O., Berger, K. L. & Bozoki, A. C. Alzheimer's disease and amnestic mild cognitive impairment weaken connections within the default-mode network: a multi-modal imaging study. *J Alzheimers Dis* **34**, 969–984 (2013).

77. Leow, A. *et al.* Impaired inter-hemispheric integration in bipolar disorder revealed with brain network analyses. *Biol Psychiatry* **73**, 183–193 (2013).

78. Schmidt, S. A., Akrofi, K., Carpenter-Thompson, J. R. & Husain, F. T. Default mode, dorsal attention and auditory resting state networks exhibit differential functional connectivity in tinnitus and hearing loss. *PLoS One* **8**, e76488 (2013).

79. Burianová, H., Ciaramelli, E., Grady, C. L. & Moscovitch, M. Top-down and bottom-up attention-to-memory: mapping functional connectivity in two distinct networks that underlie cued and uncued recognition memory. *Neuroimage* **63**, 1343–1352 (2012).

80. Yin, X. *et al.* Anatomical substrates of the alerting, orienting and executive control components of attention: focus on the posterior parietal lobe. *PLoS One* **7**, e50590 (2012).

81. Vasconcelos, L. G. *et al.* The thickness of posterior cortical areas is related to executive dysfunction in Alzheimer's disease. *Clinics* **69**, 28–37 (2014).

82. Xu, P. *et al.* Different topological organization of human brain functional networks with eyes open versus eyes closed. *Neuroimage* **90**, 246–255 (2014).

83. Sestieri, C., Capotosto, P., Tosoni, A., Luca Romani, G. & Corbetta, M. Interference with episodic memory retrieval following transcranial stimulation of the inferior but not the superior parietal lobule. *Neuropsychologia* **51**, 900–906 (2013).

84. Fabbri, S., Strnad, L., Caramazza, A. & Lingnau, A. Overlapping representations for grip type and reach direction. *Neuroimage* **S1053–8119**, 00162–1 (2014).

85. Cavanna, A. E. & Trimble, M. R. The precuneus: a review of its functional anatomy and behavioural correlates. *Brain* **129**, 564–583 (2006).

86. Sheline, Y. I. & Raichle, M. E. Resting state functional connectivity in preclinical Alzheimer's disease. *Biol Psychiatry* **74**, 340–347 (2013).

87. Delli Pizzi, S. *et al.* Structural alteration of the dorsal visual network in DLB patients with visual hallucinations: a cortical thickness MRI study. *PLoS One* **9**, e86624 (2014).

88. Collignon, O. *et al.* Impact of blindness onset on the functional organization and the connectivity of the occipital cortex. *Brain* **136**, 2769–2783 (2013).

89. Gaetz, W., Roberts, T. P., Singh, K. D. & Muthukumaraswamy, S. D. Functional and structural correlates of the aging brain: relating visual cortex (V1) gamma band responses to age-related structural change. *Hum Brain Mapp* **33**, 2035–2046 (2012).

90. Bedny, M., Pascual-Leone, A., Dravida, S. & Saxe, R. A sensitive period for language in the visual cortex: distinct patterns of plasticity in congenitally versus late blind adults. *Brain Lang* **122**, 162–170 (2012).

91. Boldt, R. *et al.* Spatial variability of functional brain networks in early-blind and sighted subjects. *Neuroimage* **S1053–8119**, 00219–5 (2014).

92. Aminoff, E. M., Kveraga, K. & Bar, M. The role of the parahippocampal cortex in cognition. *Trends Cogn Sci* **17**, 379–390 (2013).

93. Killian, N. J., Jutras, M. J. & Buffalo, E. A. A map of visual space in the primate entorhinal cortex. *Nature* **491**, 761–764 (2012).

94. Semenza, C. Naming with proper names: the left temporal pole theory. *Behav Neurol* **24**, 277–284 (2011).

95. Olson, I. R., Plotzker, A. & Ezzyat, Y. The Enigmatic temporal pole: a review of findings on social and emotional processing. *Brain* **130**, 1718–1731 (2007).

96. Hirabayashi, T. & Miyashita, Y. Computational principles of microcircuits for visual object processing in the macaque temporal cortex. *Trends Neurosci* **37**, 178–187 (2014).

97. Sabatinelli, D. *et al.* Emotional perception: meta-analyses of face and natural scene processing. *Neuroimage* **54**, 2524–2533 (2011).

98. Tompa, T. & Sáry, G. A review on the inferior temporal cortex of the macaque. *Brain Res Rev* **62**, 165–182 (2010).

99. Noonan, K. A., Jefferies, E., Visser, M. & Lambon Ralph, M. A. Going beyond inferior prefrontal involvement in semantic control: evidence for the additional contribution of dorsal angular gyrus and posterior middle temporal cortex. *J Cogn Neurosci* **25**, 1824–1850 (2013).

100. Rodrigo, M. J., Padrón, I., de Vega, M. & Ferstl, E. C. Adolescents' risky decision-making activates neural networks related to social cognition and cognitive control processes. *Front Hum Neurosci* **8**, 60 (2014).

101. Hayashi, A. *et al.* Dissociable neural systems for moral judgment of anti- and pro-social lying. *Brain Res* **1556**, 46–56 (2014).

102. Kilian-Hütten, N., Valente, G., Vroomen, J. & Formisano, E. Auditory cortex encodes the perceptual interpretation of ambiguous sound. *J Neurosci* **31**, 1715–1720 (2011).

103. Woods, D. L. & Alain, C. Functional imaging of human auditory cortex. *Curr Opin Otolaryngol Head Neck Surg* **17**, 407–411 (2009).

104. Simon, E. *et al.* Morphometry and localization of the temporal transverse Heschl's gyrus in magnetic resonance imaging: a guide for cortical stimulation of chronic tinnitus. *Surg Radiol Anat* **35**, 115–124 (2013).

105. Bassett, D. S., Brown, J. A., Deshpande, V., Carlson, J. M. & Grafton, S. T. Conserved and variable architecture of human white matter connectivity. *Neuroimage* **54**, 1262–1279 (2011).

106. Cammoun, L. *et al.* Mapping the human connectome at multiple scales with diffusion spectrum MRI. *J Neurosci Methods* **203**, 386–397 (2012).

107. Tuch, D. S. Q-ball imaging. *Magnetic Resonance in Medicine* **52**, 1358–1372 (2004).

108. Yeh, F. C., Wedeen, V. J. & Tseng, W. Y. Generalized q-sampling imaging. *Medical Imaging, IEEE Transactions on* **29**, 1626–1635 (2010).

109. Freeman, L. A set of measures of centrality based on betweenness. *Sociometry* **40**, 35–41 (1977).

110. Brandes, U. A faster algorithm for betweenness centrality. *Journal of Mathematical Sociology* **25**, 163–177 (2001).

111. Guimerà, R. & Amaral, L. A. Cartography of complex networks: modules and universal roles. *J Stat Mech* **2005**, P02001 (2005).

112. Rubinov, M. & Sporns, O. Complex network measures of brain connectivity: uses and interpretations. *Neuroimage* **52**, 1059–1069 (2010).
